# Evolutionary transfer learning enables organism-wide inference of mammalian enhancer landscapes

**DOI:** 10.64898/2026.04.07.717039

**Published:** 2026-04-08

**Authors:** Chengxiang Qiu, Riza M. Daza, Ian C. Welsh, Rupali P. Patwardhan, Beth K. Martin, Tony Li, Shizhao Yang, Niklas Kempynck, Megan L. Taylor, Olivia Fulton, Truc-Mai Le, Diana R. O’Day, Jean-Benoît Lalanne, Silvia Domcke, Stephen A. Murray, Stein Aerts, Cole Trapnell, Jay Shendure

**Affiliations:** 1. Department of Genome Sciences, University of Washington, Seattle, WA, USA; 2. Department of Molecular and Systems Biology, Dartmouth College, Hanover, NH, USA; 3. Seattle Hub for Synthetic Biology, Seattle, WA, USA; 4. The Jackson Laboratory, Bar Harbor, ME, USA; 5. Laboratory of Computational Biology, VIB Center for AI & Computational Biology (VIB.AI), Leuven, Belgium; 6. VIB-KU Leuven Center for Neuroscience, Leuven, Belgium; 7. Department of Human Genetics, KU Leuven, Leuven, Belgium; 8. Brotman Baty Institute for Precision Medicine, Seattle, WA, USA; 9. Département de Biochimie et Médecine Moléculaire, Université de Montréal, Montréal, QC, Canada; 10. Department of Molecular Life Sciences, University of Zurich, Zurich, Switzerland; 11. Institute of Stem Cell and Regenerative Medicine, University of Washington, Seattle, WA, USA; 12. Allen Discovery Center for Cell Lineage Tracing, Seattle, WA, USA; 13. Howard Hughes Medical Institute, Seattle, WA, USA

## Abstract

Understanding and modeling how the human genome encodes gene regulatory programs for thousands of cell types remains a central challenge in genomics and machine learning. However, most human cell types emerge during embryonic, fetal, and pediatric development which are inaccessible to comprehensive molecular profiling. To overcome this, we hypothesized that the mismatch in evolutionary rates between *cis*-acting enhancers (fast) and the *trans*-acting regulatory programs that interpret them (slow) creates an opportunity for ‘evolutionary transfer learning’. Specifically, models trained to predict cell type-specific enhancers in one species should generalize to the orthologous cell types and enhancers of related species. To test this, we generated a single-cell atlas of chromatin accessibility spanning mouse embryonic day 10 (E10) to birth (P0). Using combinatorial indexing^1^, we profiled 3.9 million nuclei from 36 staged embryos, resolving genome-wide accessibility in 36 cell classes and 140 cell types. With the goal of identifying distal enhancers for all cell classes, we trained a series of multi-output deep learning models (CREsted^2^), each addressing limitations of the preceding approach. An ‘evolution-naive’ model achieves strong performance on heldout peaks, but exhibited two failure modes during genome-wide inference: overprediction at tandem repeats and conflation of promoter and distal enhancer grammars. An ‘evolution-aware’ model resolves these by regrouping accessible regions based on functional coherence across syntenic orthologs, but fails to generalize across species — suggesting insufficient sequence diversity during training. Finally, STEAM (Synteny-aware Transfer learning for Enhancer Activity Modeling), our ‘evolution-augmented’ model, expands the training corpus to include enhancer orthologs from up to 241 mammalian genomes (Zoonomia^3^) in a synteny-supervised manner. This increases the effective data scale by up to 195-fold, markedly improving generalization across mammals despite greater label noise. We apply STEAM predict enhancers for all major developmental lineages throughout the human, mouse (*HumMus*) and 239 additional mammalian genomes^3^ (*BabaGanoush*), *i.e.* 32 × 241 = 7,712 genome-wide enhancer tracks. Together, our results unify advances in single-cell profiling, deep learning, and comparative genomics into a framework for the evolutionary transfer learning of noncoding regulatory grammars. More broadly, our work supports the view that model organisms and evolutionarily diverse genomes are indispensable resources for accelerating the AI-enabled exploration of human biology.

## INTRODUCTION

Recent breakthroughs in protein structure prediction, exemplified by AlphaFold^5^ and RoseTTAFold^6^, are often credited to the synergy of transformer architectures, large-scale computation, and a growing repository of atomic-resolution structures^7^. But the most indispensable ingredient was arguably evolution. Protein orthologs can diverge dramatically in primary amino acid sequence while maintaining a nearly identical structure and function, and protein orthologs can functionally complement one another across vast evolutionary distances (*e.g.* yeast ↔ human)^8^. This mismatch — sequence evolves fast, structure-function evolves slowly — is the supervisory signal that made high-fidelity structure prediction possible. Modern structure predictors achieved their accuracy only once densely populated multiple sequence alignments (MSAs) from genome and metagenome sequencing, generated in abundance by the genomics community for unrelated goals, allowed them to extract co-evolutionary couplings that no amount of structural data alone could provide^9,10^.

Surprisingly, no equivalent principle has been systematically exploited in regulatory genomics. Despite a decade of deep learning in this field^11,12^, nearly all major efforts lack an evolutionary dimension (with some exceptions^13–18^). Models such as Borzoi, BPnet, Enformer, Basenji and AlphaGenome predict biochemical readouts from DNA sequence with impressive fidelity, yet are trained on a single reference genome and a static set of molecular profiles^19–23^. A parallel class of DNA models (*e.g.* Evo, Nucleotide Transformer, GPN) draws on multi-species corpora during pretraining, internalizing evolutionary statistics implicitly^24–27^, analogous to how ESMFold^28^ learns co-evolution from protein sequence alone, without an MSA at inference. But whether implicit exposure substitutes for principled comparative supervision remains an open question.

We reasoned that an analogous mismatch exists in gene regulation: *cis*-regulatory elements evolve rapidly, but the *trans*-acting programs that interpret them (*i.e.* cell type-specific transcription factor repertoires) evolve slowly. A striking demonstration comes from mice engineered to carry human chromosome 21^29^. Hepatocytes from these animals almost perfectly reproduced the epigenomic and transcriptomic signatures of the chromosome in human hepatocytes^30–35^. Put simply, the *trans*-acting milieus of mammalian cell types are ancient and stable, whereas the *cis*-regulatory landscape rewires continuously.

Why not simply learn human regulatory grammars on human data? First, data availability: nearly all human cell types arise during embryonic, fetal, and pediatric stages that are largely inaccessible to deep molecular profiling. A comprehensive human epigenomic atlas — *i.e.* from zygote to embryo to fetus to child to adult, at whole-body scale and high temporal resolution — may never be obtainable. In contrast, we and others have built high-resolution, whole-animal, single-cell atlases of zebrafish^36–38^, mouse^39–43^ and rhesus macaque^44^. Given that cell type-specific enhancer grammars are far more conserved than the genome sequences on which they act, models trained on such atlases and applied to human sequence could provide ‘virtual access’ to *in vivo* human regulatory landscapes that are otherwise off-limits.

A second motivation is statistical. Single-species models are limited not just by the number of regulatory elements in a genome, but also by restricted sequence diversity. Even if the full human enhancer catalog were known, the number active in any given cell type may be insufficient to learn high-dimensional grammars involving combinatorial motif syntax, positional preferences, and long-range dependencies. Orthologous enhancers provide a natural solution: sequence variation under shared functional constraint. Because purifying selection on enhancers is weak at the nucleotide level, their orthologs frequently encode highly divergent yet functionally equivalent sequences^45,46^. And although excessive divergence can prevent alignment, the initial human-mouse genome comparison showed that syntenic regions — identical by descent, even if neutrally evolving — remain alignable at ~0.50 substitutions per position^47^, while other studies have shown functional complementation of syntenic enhancers across even greater phylogenetic distances (*e.g.* mammals ↔ chicken or zebrafish^45,46^). In this sense, synteny provides the critical bridge between sequence divergence and functional conservation, allowing evolutionarily related, functionally conserved elements to be aligned even when sequence similarity has heavily eroded. Indeed, the genomes of >6,500 extant mammals collectively contain billions of orthologous developmental enhancers — an opportunity to expand the training corpus by orders of magnitude. Properly harnessed, this phylogenetic breadth should yield gene regulatory models with greater accuracy, generalizability, and interpretability.

Here, we exploit this evolutionary-rate contrast to learn dozens of cell type-specific regulatory grammars that transfer across mammalian genomes, including the human genome. We first generated a single-cell, whole-animal atlas of chromatin accessibility spanning mouse embryonic day 10 to birth, resolving 36 cell classes and 140 cell types across 3.9 million nuclei. Using CREsted^2^, we trained an evolution-naive sequence model that exposed key failure modes in genome-wide inference. An evolution-aware successor resolved these by using concordance across syntenic orthologs to isolate sequence-intrinsic enhancer activity. Finally, we developed STEAM (Synteny-aware Transfer learning for Enhancer Activity Modeling), which augments training with synteny-anchored enhancer orthologs from up to 240 mammalian genomes from the Zoonomia project^3^, a 195-fold expansion of the effective corpus, to learn grammars that generalize across mammals. Although our initial goal was predicting human enhancer landscapes, the resulting framework supports inference across mammalian genomes generally: an unanticipated but natural consequence of training on phylogenetic breadth.

## RESULTS

### Single-nucleus chromatin accessibility profiling of whole mouse embryos from E10 to P0

**Figure 1.**
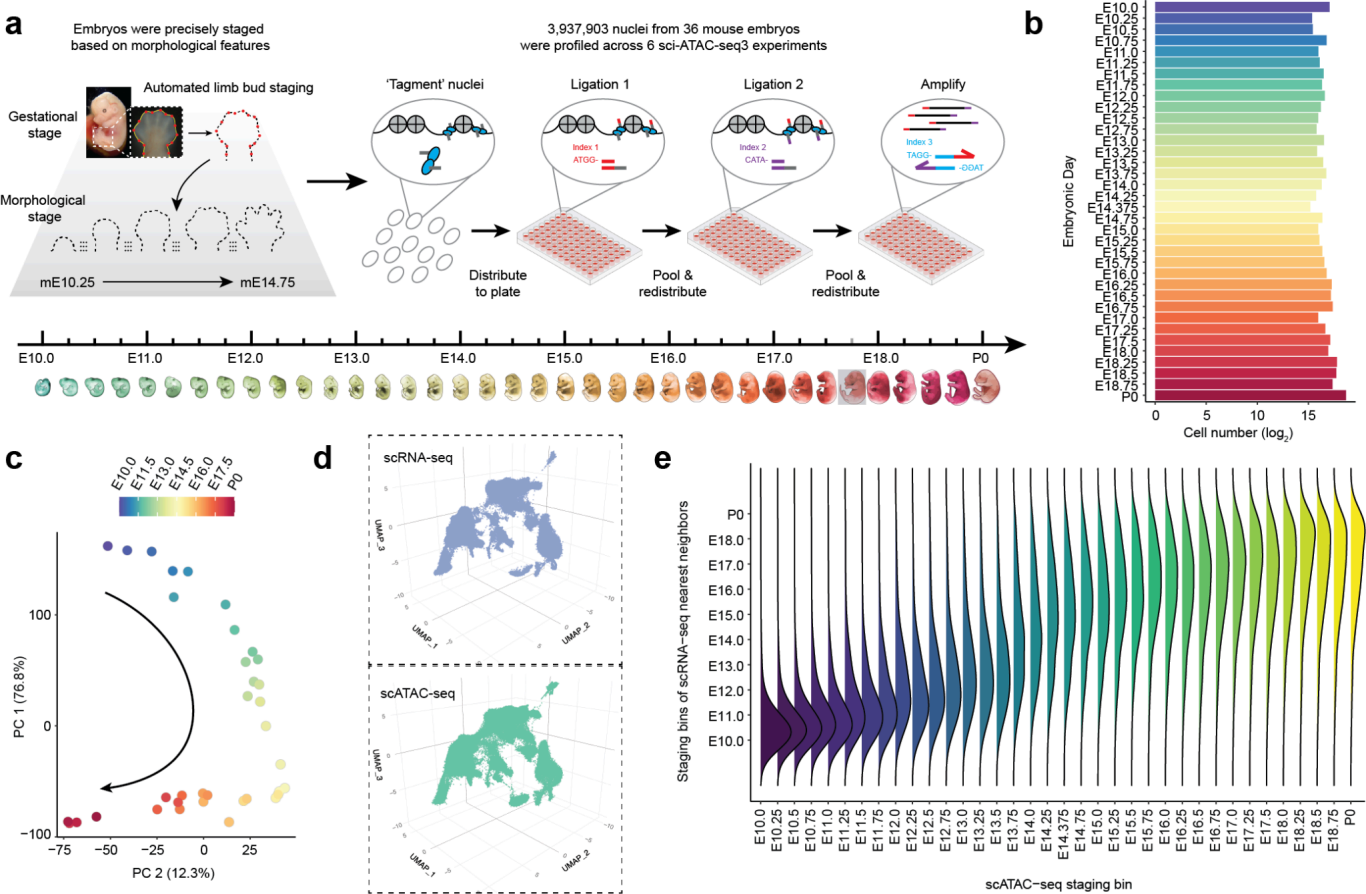
A single-cell chromatin-accessibility timelapse of mouse development from E10 to P0. **a,** Embryos were collected and staged based on morphological features, including an automated method leveraging limb bud geometry for E10–E15^40^. We selected 36 embryos for sci-ATAC-seq3, exactly one per 6-hour bin from E10 to P0 with the exception of E17.75, which remains missing from the series (gray box). Across six experiments, we profiled chromatin accessibility in 3.9 million cells from 36 embryos. The staging diagram and sci-ATAC-seq3 workflow were adapted from Domcke et al.^51^ and Qiu et al.^40^. **b,** The log_2_ scaled number of cells profiled per embryo, sorted by staging bin. **c,** Embeddings of pseudo-bulk ATAC-seq profiles of 36 embryos in PCA space, plotting PC1 vs. PC2. Briefly, single cell profiles were downsampled to a uniform number per embryo, aggregated to 36 pseudo-bulk profiles, and subjected to dimensionality reduction (PCA). Embryos are colored by staging bin; the arrow highlights developmental progression. **d,** 3D UMAP visualization of co-embedded cells from published scRNA-seq^40^ and scATAC-seq (this study) data. From each dataset, we selected 15,000 cells from each of 36 shared staging bins, and then performed integration with scGLUE^50^. The same UMAP is shown twice, highlighting cells from either scRNA-seq (top) or scATAC-seq (bottom) data. **e,** For scATAC-seq cells from each staging bin (*x*-axis), the density of staging-bins-of-origin of their nearest scRNA-seq derived neighbors (n = 10) in the scGLUE-derived co-embedding is plotted (*y*-axis). The density curves on the *y*-axis, generated by geom_density_ridges, were smoothed using a bandwidth of 0.6. P0 was treated as E19.0 for this analysis.

For molecular profiling of mammalian cell type emergence and diversification at whole-organism scale, we focused on *Mus musculus* (mouse). In a recent study, we collected and ontogenetically staged 523 mouse embryos spanning E8 to P0^40^. We then applied scRNA-seq to profile 11.4 million nuclei from 74 of these embryos, spaced at 2- to 6-hour intervals. For this study, we selected 36 embryos from this same staged set, spaced at 6-hour intervals from E10 to P0, and extracted nuclei (Fig. 1a; Supplementary Table 1; E17.75 bin absent). We preferentially selected female embryos to minimize sex-linked mapping artifacts^48^ (29 female; 7 male) and processed whole animals without tissue dissection, as in our prior work^40–42,49^

Six sci-ATAC-seq3 experiments were conducted across defined stage ranges (E10–E13.75 × 3; E14–E15.75; E16–E18; E18.25–P0). Following sequencing, demultiplexing, embryo assignment, mapping to the mm10 reference genome, and duplicate removal (avg. 24%; Supplementary Table 2), peaks were called per embryo and merged into a master set of 376,574 peaks. After aggressive filtering of low-quality nuclei and doublets (Supplementary Fig. 1a), the final matrix comprised 3,937,903 cells × 376,574 peaks, averaging 109,386 cells per embryo (median 90,804; range 37,287–420,085; Fig. 1b). Median unique reads per cell was 3,922 (35.7% overlapping peaks; 15.0% within 1 kb of a transcription start site (TSS)). Of note, we observed modest shifts in fragment size distributions across timepoints and mild experiment-level batch structure across pseudobulk profiles (Supplementary Figs. 1b, 2a); while these may reflect a mixture of biological and technical factors, we did not detect any substantial influence on clustering, trajectory structure, or model behavior.

Upon performing principal components analysis (PCA), pseudobulked ATAC profiles showed top PCs strongly correlated with developmental time (PC1+PC2 = 89%; Fig. 1c) and high correlation between neighboring timepoints (Supplementary Fig. 2a). Integration of downsampled scATAC-seq and scRNA-seq matrices (540,000 cells each; 15,000 cells × 36 timepoints) via scGLUE^50^ confirmed that nearest neighbors largely originated from matched or neighboring timepoints (Figs. 1d,e), thereby validating data quality.

### Iterative annotation of 13 cell lineages, 36 cell classes and 140 cell types

**Figure 2.**
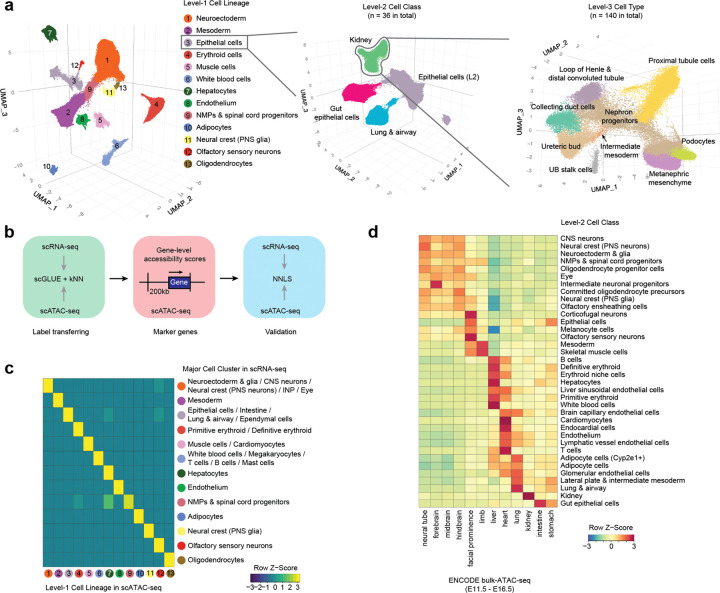
Iterative annotation of a single cell mouse developmental chromatin accessibility atlas. **a,** Left: 3D UMAP visualization of 3,937,903 single cell chromatin accessibility profiles, colored by Level-1 annotation. Middle & Right: Three rounds of annotation were performed through dimensionality reduction, clustering and label transfer, illustrated by epithelial cells (Level-1) → kidney (Level-2) → various renal epithelial cell types (Level-3). **b,** To annotate cell lineages, classes or types, we applied a *k*-NN heuristic to an scGLUE^50^-derived co-embedding with scRNA-seq data from matching timepoints (Fig. 1d). Next, we manually refined these annotations by verifying that marker genes for each cell lineage exhibited high gene-level accessibility scores, calculated by counting reads overlapping gene bodies (plus upstream 200 kb). Finally, we validated these annotations by performing non-negative least squares (NNLS) regression between gene-level accessibility scores from scATAC-seq and gene expression levels from scRNA-seq^42^. **c,** Heatmap of combined regression coefficients (NNLS; row-scaled) for 13 major scRNA-seq cell clusters^40^ (rows) vs. 13 Level-1 scATAC-seq cell lineages (columns; annotation key in panel **a**). CNS: central nervous system. PNS: peripheral nervous system. INP: intermediate neuronal progenitors. NMPs: Neuromesodermal progenitors. **d,** Overlap of 36 peak sets from Level-2 scATAC-seq cell classes (rows) vs. 12 peak sets from bulk ATAC-seq of dissected mouse embryonic tissues by ENCODE^63^. Briefly, peaks identified by ENCODE for each tissue were merged across timepoints (E11.5–E16.5). We also downsampled each Level-2 cell class to 10,000 cells, recalled peaks, calculated their specificity scores using Jensen-Shannon divergence, and selected the 2,000 most specific peaks. For each Level-2 cell class peak set × ENCODE tissue peak set, we counted the number of base pairs overlapping base pairs. Finally, we performed row-based normalization to account for total length, and plotted the resulting Z-scores.

We annotated these 3.9 million scATAC-seq profiles via three iterative rounds of dimensionality reduction, clustering, and label transfer. For the first round, we applied BPCells^52^ to perform latent semantic indexing (LSI)-based dimensionality reduction, embedded cells in 3D UMAP space, and used Louvain clustering to define major cell lineages (Fig. 2a). Labels were transferred from our matched scRNA-seq atlas^40^ via a *k*-nearest neighbors (*k*-NN) heuristic applied to the scGLUE co-embedding (Fig. 1d), and then manually refined based on marker gene accessibility scores (Fig. 2b; Supplementary Table 3).

This first round yielded 13 cell lineages (Level-1): neuroectoderm, mesoderm, epithelial cells, erythroid cells, muscle cells, white blood cells, hepatocytes, endothelium, NMPs & spinal cord progenitors, adipocytes, neural crest (PNS glia), olfactory sensory neurons, and oligodendrocytes (Fig. 2a–c). Lineage compositions were highly consistent between scATAC-seq and scRNA-seq modalities (Supplementary Fig. 2b–c), and Level-1 annotations were robust to the choice of features for dimensionality reduction (Supplementary Fig. 2d)^53,54^. Two additional rounds of iterative subclustering yielded 36 cell classes (Level-2) and 140 cell types (Level-3) (Fig. 2a; Supplementary Figs. 3–5). We note that these annotations are preliminary and may be refined as we and others further analyze the data.

Of 204 cell types previously annotated in our scRNA-seq atlas^40^, 114 mapped one-to-one to 114 Level-3 annotations, 56 mapped many-to-one to 24 Level-3 annotations (reflecting lower resolution in scATAC-seq data, *e.g.* diencephalon/midbrain/MHB → midbrain), and 34 were absent, either because they predate E10 (*e.g.* notochord) or are too rare to detect (<0.25% of the scRNA-seq dataset, which profiled three times as many cells) (Supplementary Fig. 5; Supplementary Table 4). Conversely, 138 of 140 Level-3 scATAC-seq cell types had at least one scRNA-seq counterpart. One exception was ureteric bud stalk cells, present in the scRNA-seq atlas but not resolved as a distinct cluster (Supplementary Fig. 6a–c). The second was a population we term ‘erythroid niche cells’, absent from the scRNA-seq atlas entirely (Supplementary Fig. 6d–e). These cells cluster with the yolk sac-derived primitive erythroid lineage but are distinguished by a delayed temporal profile persisting through P0 (Supplementary Fig. 6f), markedly reduced accessibility at embryonic globin loci (*Hba-x*, *Hbb-y*), and increased accessibility at lysosomal (*Lamp1*, *Ctsd*, *Ctsb*, *Ctsl*, *Hexa*), heme catabolism (*Hmox1*), and erythropoiesis-related cytokine genes (*Epo*, *Csf2ra*) (Supplementary Fig. 6g; Supplementary Table 5)^55–61^. Their absence from scRNA-seq data could reflect a transcriptionally low-output state, though an alternative is that they represent primitive pyrenocytes — extruded nuclei of enucleating primitive erythrocytes^62^. We favor the former interpretation, as pyrenocytes would be expected to lack active chromatin remodeling machinery, difficult to reconcile with the coordinated accessibility gains observed, together with robust quality metrics for this population (Supplementary Fig. 6h).

To recover accessible regions specific to rarer cell types that may be absent from the initial master peak set, we re-called and merged peaks from pseudobulked profiles at each annotation level, yielding 695,910 (495 Mb), 795,998 (583 Mb), and 793,816 (568 Mb; 21% of mm10) peaks at Levels 1, 2, and 3, respectively. As expected, these annotation-aware peak sets overlap substantially, *e.g.* 89% and 77% of Level-3 intervals are also spanned by Level-2 and Level-1 intervals, respectively. Reassuringly, they also show extensive overlap with peaks identified by bulk ATAC-seq of mouse embryonic tissues by the ENCODE Consortium^63^ (Fig. 2d).

These single cell profiles, peak sets and corresponding annotations constitute a whole-organism map of chromatin accessibility, sampled at 6-hour resolution from the onset of organogenesis (E10) through birth (P0) in the mouse, the most widely used mammalian model of human biology. Together with our matched scRNA-seq atlas from equivalently staged embryos^40^, they provide a powerful resource for modeling how a mammalian genome encodes and deploys *cis*-regulatory programs as cell types emerge and diversify during development.

### How well do models of cell-class-specific accessibility perform on genome-wide inference?

**Figure 3.**
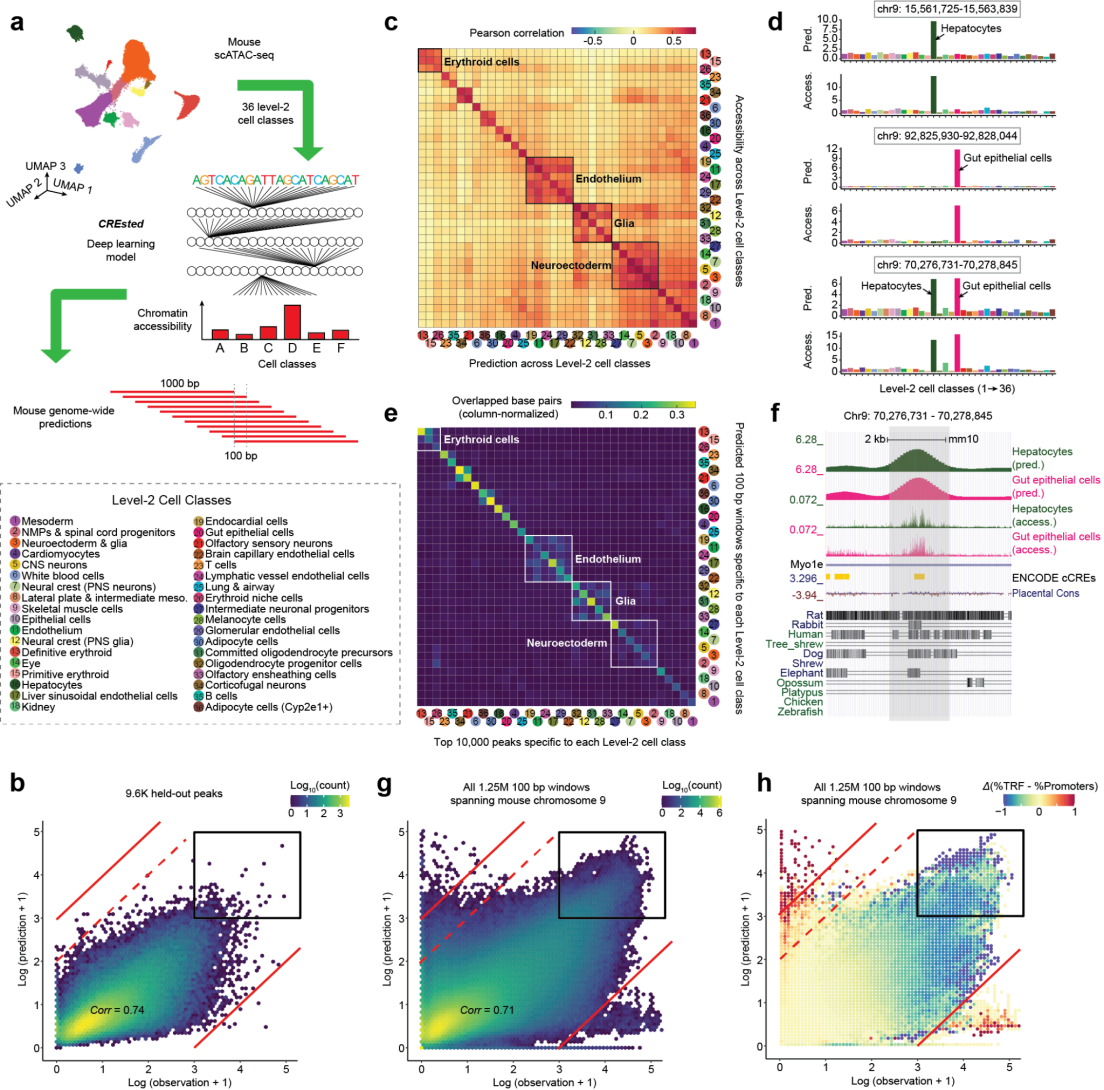
Evolution-naive modeling of chromatin accessibility across 36 Level-2 cell classes. **a,** We trained CREsted^2^, a multi-output convolutional neural network framework that predicts quantitative chromatin accessibility from DNA sequence, on the mouse scATAC-seq timelapse atlas. For evolution-naive modeling, we trained on Level-2 cell class peaks and then sought to make genome-wide predictions. For this, the mouse genome was segmented into sliding 1,000 bp windows, and predictions averaged to 100-bp resolution. Candidate 100-bp windows for each cell class were identified by requiring both a Z-transformed predicted score ≥ 3 and a Z-transformed specificity ≥ 3. **b,** Scatterplots of observed (*x*-axis; normalized Tn5 cut-site counts; log-n scaled) vs. predicted (*y*-axis; evolution-naive CREsted model; log-n scaled) chromatin accessibility across 9,583 peaks held out during fine-tuning, shown for all 36 Level-2 cell classes and colored by log_10_ scaled density. Solid red lines demarcate grossly overpredicted (above *y* > *x* + 3) and grossly underpredicted (below *y* < *x* - 3) windows. A dotted red line marks moderately overpredicted windows (above *y* > *x* + 2). The black rectangle highlights windows that are both predicted and observed to be highly accessible (*x* > 3 && *y* > 3). The same data, stratified by cell class, are shown in Supplementary Fig. 7. **c,** Pairwise Pearson correlations were computed among the 36 Level-2 cell classes between normalized Tn5 cut-site counts from scATAC-seq data (rows) and model predictions (columns) for 9,583 held-out peaks. Correlation coefficients are shown in the heatmap, with rows and columns ordered by hierarchical clustering. The black rectangles highlight subsets of related cell classes exhibiting elevated cross-correlation. Cell class labels are shown to the left of panel **e**. **d,** Observed (top) and predicted (bottom) chromatin accessibility is plotted for each of the 36 Level-2 cell classes, for three representative regions from the test set, including two regions exhibiting accessibility in one cell class, and one region exhibiting accessibility in two related cell classes. In all three cases, we observe strong agreement between observed and predicted accessibility. **e,** For each 100-bp bin, we averaged predictions from overlapping windows to obtain a 36-dimensional accessibility vector, and computed cell-class specificity scores using a softmax-based metric (Supplementary Fig. 8a–b). Plotted here are pairwise overlaps (bp) between each class’s 10,000 peaks used for initial training (columns) and 100-bp windows with both high predicted accessibility and specificity (rows; both Z-transformed scores ≥ 3; Methods), column-normalized by the total size of each class’s training peaks. Cell class labels are shown to the left. **f,** Genome browser view of predicted (top two rows; evolution-naive CREsted model) and observed (normalized Tn5 cut-site counts) chromatin accessibility for the gut epithelial and hepatocyte cell classes, together with ENCODE cCRE, placental conservation and synteny tracks (bottom three rows). This example corresponds to the bottommost example highlighted in panel **d**. Generated using the UCSC Genome Browser (mm10). **g,** Same as panel **b**, but across 1.25M × 100 bp windows spanning mouse chr. 9 for all 36 Level-2 cell classes. **h,** Same as panel **g,** but colored by the difference between the percentage of windows overlapping tandem repeats and the percentage overlapping promoters (−1,500 bp to +500 bp relative to TSSs). The scale is oriented such that red indicates tandem repeat enrichment and blue indicates promoter enrichment. Gross overpredictions are highly enriched for tandem repeats (top left of plot), while very highly accessible observations/predictions are highly enriched for promoters (black rectangle).

We next sought to use this whole-organism atlas spanning mouse development to model cell-class-specific chromatin accessibility with CREsted, a framework for decoding enhancers through sequence-to-function deep learning^2^. A key attribute of CREsted is its ability to jointly learn and predict quantitative chromatin accessibility from DNA sequence across many cell types within a single model (Fig. 3a).

We first trained an ‘evolution-naive’ CREsted model to predict chromatin accessibility at candidate regulatory regions across 36 cell classes. After excluding promoter proximal peaks from the Level-2 peak set (± 2.5 kb of annotated TSS), we trained on 299,281 × 2,114-bp windows (CREsted’s input length^2^) centered on the 10,000 most specific peaks per class, followed by fine-tuning on 106,813 windows centered on the 3,000 most specific peaks per class. For both stages, peaks were split into training (chrs. 1–7, 11–17, 19, X, Y), validation (chrs. 8, 10), and test (chrs. 9, 18) sets. The resulting model exhibited strong performance on held-out peaks (Pearson’s *r* = 0.74, range = 0.63–0.82 across 36 cell classes) (Fig. 3b; Supplementary Fig. 7). Cross-correlation analysis revealed largely cell-class-specific signals alongside coherent blocks corresponding to closely related lineages (Fig. 3c). Inspection of specific loci found that the model accurately predicted both cell class-specific peaks and peaks shared across related lineages (Fig. 3d).

Most sequence-based models of regulatory activity are trained and evaluated on predefined sets of candidate enhancers^12,20,22^, as we do above. However, whether such models can be meaningfully applied beyond these curated regions has received comparatively little attention. This omission may be consequential: strong performance within a training distribution does not guarantee that a model has learned regulatory logic that generalizes across the rest of the genome, let alone across to the genomes of other species. To assess this, we tiled the mouse reference genome with overlapping 1-kb windows (100-bp step size; predictions averaged across overlapping windows) and applied the ‘evolution-naive’ model, yielding 36 genome-wide tracks of predicted accessibility and specificity for 28M × 100 bp windows spanning mm10 (Supplementary Fig. 8a–b; Supplementary File 1).

At a coarse level, we recovered cell class-specific regulatory landscapes. Highly scoring windows were enriched for overlap with distal peaks used during training and recapitulated observed accessibility patterns at representative loci (Fig. 3e–f). However, while overall performance remained comparable to held-out peaks (Pearson’s *r* = 0.71 for held-out chr. 9), scatterplots revealed numerous windows that were grossly overpredicted (log-n[predicted/observed] > 3) or underpredicted (log-n[predicted/observed] < −3) (Fig. 3g). Gross overpredictions were strongly enriched for tandem repeats (OR: 24-fold), (Fig. 3h; Supplementary Fig. 8c–d). We speculate this reflects motif-like matches within repeating units that mimic enhancer grammar without corresponding regulatory activity. Such false positives are highly problematic for genome-wide inference, as tandem repeats and other low complexity sequences appear frequently throughout the genome.

A second failure mode relates to promoters, which are among the most highly accessible regions in the genome, yet whose sequences encode little of the regulatory grammar underlying cell type-specific expression. Housekeeping promoters are constitutively accessible across cell types, while cell type-specific promoter accessibility reflects the activity of distal enhancers acting in *trans*, rather than specificity encoded in the promoters themselves. Despite our exclusion of peaks within 2.5 kb of annotated TSSs, the most highly accessible observations and predictions were highly enriched for promoters (OR: 11.0-fold for windows with log-n[predicted] > 3 and log-n[observed] > 3; Fig. 3g–h). Although technically accurate — these regions are indeed highly accessible — this conflation is problematic, as our broad objective is to learn distal enhancer grammars rather than raw accessibility, and promoter masking was intended to enforce this. We interpret this as evidence that the evolution-naive model learned a mixed grammar spanning both distal enhancers and promoters, possibly because many promoters are unannotated, ambiguously annotated, or otherwise not captured by simple distance-to-TSS exclusion.

### Evolutionary coherence isolates distal enhancer grammars and facilitates genome-wide inference

To summarize, although the evolution-naive CREsted model generalized well across most of the genome, a substantial fraction of its highest-scoring predictions reflected non-enhancer signal, including tandem repeats and promoters. These failure modes are problematic for genome-wide inference. Tandem repeats generate large numbers of false positives, while promoters — despite being genuinely accessible — encode regulatory grammars that are largely shared across cell types and therefore confound our goal of learning cell-class-specific distal enhancer grammar. To mitigate these confounders, we reasoned that most bona fide distal enhancers should satisfy three criteria. First, they should be syntenically retained across many mammalian genomes. Second, their orthologs should exhibit ‘evolutionary coherence’ — consistent predicted activity profiles across cell classes when scored by the evolution-naive model. Third, their predicted activity should not depend on tandem repeats within or nearby.

**Figure 4.**
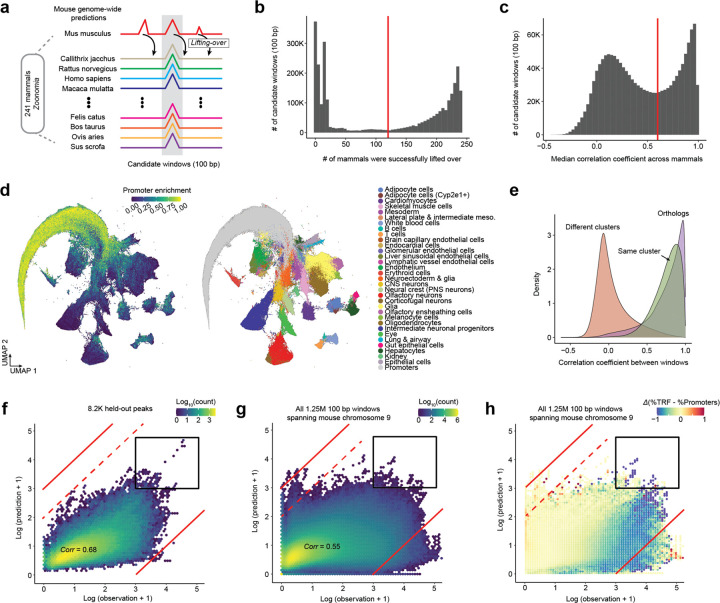
Evolutionary coherence separates distal enhancer grammar from promoter & repeat-associated signals. **a,** Schematic. We performed mouse genome-wide inference with the evolution-naive model. Windows with high predicted accessibility and specificity were filtered for evolutionary retention and coherence of model predictions across Mammalia^3^. **b,** To 2.79M windows predicted to be highly (Z ≥3) and specifically (Z ≥3) accessible in ≥1 cell class, we performed liftover to 240 other mammalian genomes^3^. 1.47M windows were successfully lifted over to ≥120 other species (red vertical line). **c,** To those 1.47M windows, we applied the evolution-naive CREsted model to syntenic regions of other mammals, and calculated the pairwise Pearson’s *r* between the resulting 36-value predicted accessibility vectors for mouse vs. ortholog. Windows exhibiting a median correlation coefficient greater than 0.6 (red vertical line) across species were retained. **d,** Predicted score matrix for the remaining 547,317 windows × 36 cell classes, subjected to UMAP embedding and Leiden clustering. 2D rendering of 3D UMAP is shown, colored by promoter enrichment (left) or annotation (right). Promoter enrichment calculated as % of a window’s 30 nearest neighbors (Euclidean) within 2.5 kb of an annotated TSS. **e,** We randomly sampled 500 windows from each of the 32 non-promoter clusters and computed Pearson’s *r* between their 36-value predicted accessibility vectors vs. those of windows from the same cell class cluster (red), different cell class cluster (green), or syntenic orthologs (purple). The resulting distributions are visualized as density plots. **f,** Scatterplots of observed (normalized Tn5 cut-site counts; log-n scaled; *x*-axis) vs. predicted (evolution-aware CREsted model & tandem repeat mitigation heuristic; log-n scaled; *y*-axis) accessibility at 8,223 held-out peaks, shown for all 32 cell classes and colored by log_10_ scaled density. Similar to Fig. 3b, which was generated with the evolution-naive model. **g,** Same as panel **f**, but across 1.25M × 100 bp windows spanning mouse chr. 9 for 32 cell classes. Similar to Fig. 3g, which was generated with the evolution-naive model and without the tandem repeat mitigation heuristic. **h,** Same as panel **f**, but across 1.25M × 100 bp windows spanning mouse chr. 9 for 32 cell classes and with points colored by the difference between % of windows overlapping tandem repeats and % overlapping promoters (−1,500 bp to +500 bp relative to TSSs). Red indicates tandem repeat enrichment. Blue indicates promoter enrichment. Similar to Fig. 3h, which was generated with the evolution-naive model and without the tandem repeat mitigation heuristic.

To operationalize the first two criteria, we applied a two-stage evolutionary filter to the genome-wide evolution-naive predictions (Fig. 4a). We began with the 2.79 million 100-bp windows (~10% of the mouse genome) scoring Z ≥ 3 for both predicted accessibility and specificity in at least one cell class. As a first filter, we lifted these windows from mm10 to the 240 Zoonomia genomes^3^. The resulting per-window liftover counts were clearly bimodal (Fig. 4b), and we retained the 1.47 million windows (53%) syntenically preserved in at least half. As a second filter, we quantified ‘evolutionary coherence’. For each retained window, we extracted 2,114 bp (CREsted input length) centered on its syntenic coordinates in each non-mouse genome (296 million sequences; mean 201 orthologs per window) and scored all orthologs with the evolution-naive model. We then correlated each mouse window’s 36-value predicted accessibility vector against each ortholog’s. The median Pearon’s *r* per window was bimodal (Fig. 4c). At a threshold of *r* ≥ 0.6, we retained 547,317 windows.

To organize these windows, we performed Leiden clustering on their 36-value predicted accessibility vectors, yielding 33 clusters: one large cluster highly enriched for promoters (190,035 windows) and 32 smaller clusters (1,018–45,655 windows) that mapped largely one-to-one to our Level-2 cell classes (Fig. 4d; Supplementary Fig. 9a). A few closely related classes were not separable and were merged (primitive & definitive erythroid → erythroid; oligodendrocyte progenitors & committed oligodendrocyte precursors → oligodendrocytes). A few others mapped to the promoter cluster and were discarded (NMPs; erythroid niche cells).

After filtering ENCODE blacklisted regions, the 32 non-promoter clusters comprised 354,450 × 100-bp windows (74,621 regions spanning 35.4 Mb after merging adjacent windows within each cluster). Notably, the distribution of pairwise coherence among mouse windows within a non-promoter cluster closely overlaps that between mouse windows and their mammalian orthologs (Fig. 4e). We next used these 32 clusters to train an ‘evolution-aware’ CREsted model. Although the promoter cluster was already excluded, we further guarded against promoter grammars confounding our training by manually zeroing out accessibility within 2.5 kb of annotated TSSs. We trained initially on the 10,000 most specific windows per cluster, then fine-tuned on the top 3,000; for both stages, chromosome splits matched the evolution-naive model.

Using the resulting ‘evolution-aware’ model, we performed genome-wide prediction as previously (Fig. 3a). Because the aforedescribed tandem repeat failure mode was driven by motif-like sequences within repeating units rather than by any coherent regulatory signal, we reasoned that predictions at such regions should be destabilized by sequence perturbation. We therefore also introduced a heuristic in which regions containing algorithmically identified tandem repeats^64^ were iteratively replaced with random sequences matched for local dinucleotide frequencies, and the mean prediction used as the final score (Supplementary Fig. 9b).

The evolution-aware model exhibited lower correlation between observed vs. predicted accessibility than the evolution-naive model on held-out peaks (*r* = 0.74 vs. 0.68; Fig. 3b vs. Fig. 4f) and genome-wide predictions (*r* = 0.71 vs. 0.55; Fig. 3g vs. Fig. 4g). This reduction might superficially appear to represent a step backward, but it largely reflects the deliberate suppression of promoter and other off-target regulatory grammars — signals that inflate correlation with observed accessibility but are not the intended targets of inference. In particular, in contrast to the evolution-naive model, the evolution-aware model substantially downweights promoter accessibility (Fig. 4g,h), eliminating the dominance of promoters among the most highly predicted regions upon genome-wide inference (black-boxed regions in Fig. 3g,h vs. Fig. 4g,h). Furthermore, we observed a marked reduction in tandem repeat-associated overprediction (upper-left quadrant of Fig. 3g,h vs. Fig. 4g,h; Supplementary Fig. 9c). Together, these results indicate that both key failure modes encountered during genome-wide inference with the evolution-naive model have been substantially mitigated.

Because we apply the evolution-aware model genome-wide rather than to a predefined peak set, raw model outputs lack an intuitive reference frame for ranking predicted enhancers across the genome. To address this, we applied a scoring rubric similar to our Phred-scaled, non-parameteric CADD scores for genome-wide variant effect prediction^65,66^. For each of the 32 cell classes, genomic windows were ranked by predicted accessibility, and for each score we defined P as the fraction of genomic windows with lower predicted values — a genome-wide percentile rank. We then converted these ranks into an Genome-wide Phred-like Score (GPS = −10 × log_10_[1 − P]), facilitating their immediate interpretation (*e.g.* GPS 30 = top 0.1% of genome-wide predictions). To call peaks, we focused on the top 100,000 × 100-bp windows per cell class (10 Mb; GPS > 24.5; 0.355% of the genome). Adjacent high-scoring windows were merged, and for each merged region we extracted a 2,114-bp sequence centered on the region (matching the model input length), whose predicted accessibility was assigned to the region. Finally, we applied a trimming heuristic to identify the ‘core region’ of each candidate developmental enhancer, defined as the smallest contiguous subsequence whose predicted accessibility is at least 90% of the full 2,114-bp score (Supplementary Fig. 9d). Across all 32 cell classes, this procedure yielded 318,423 core candidate developmental enhancers (mean ± SD: 530 ± 316 bp) whose union spans 65.4 Mb (2.3%) of the mouse reference genome (Supplementary File 2).

### Candidate developmental enhancers are conserved and predict cell type-specific gene expression

**Figure 5.**
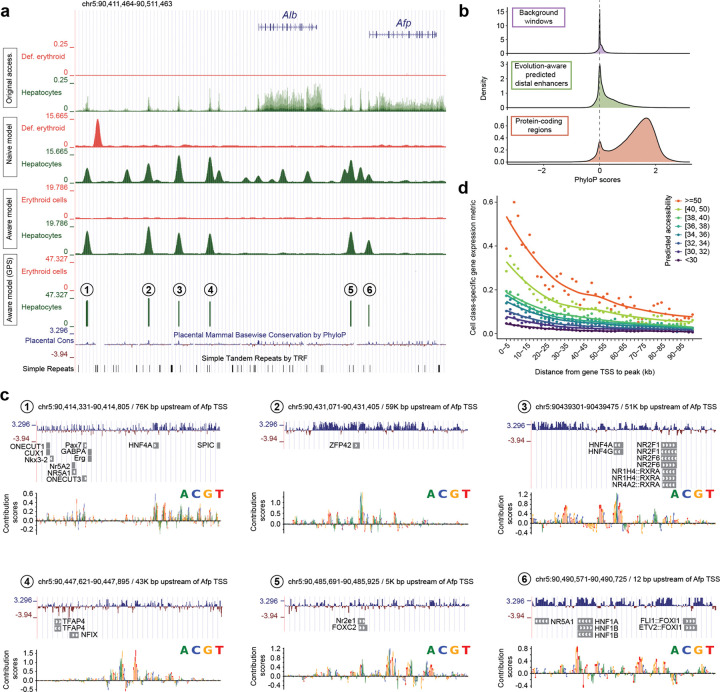
Evolution-aware modeling identifies distal enhancers that predict cell-type-specific gene expression. **a,** Genome browser view of 100 kb region encompassing *Alb* and *Afp* loci (mm10, chr5:90,411,464–90,511,463). Four pairs of definitive erythroid and hepatocyte tracks are shown (top to bottom): normalized Tn5 cut-site counts (pseudobulk), evolution-naive predicted accessibility, evolution-aware predicted accessibility, and GPS-scaled, trimmed enhancer predictions derived from the evolution-aware model. Additional annotation tracks are shown above (gene structure) and below (placental conservation, simple tandem repeats). **b,** Distribution of phyloP scores (60 placental mammals), computed by averaging nucleotide-level scores within each element, for background regions (n = 300,000 randomly selected 200-bp genomic windows; top row), evolution-aware, core-trimmed distal enhancer predictions (n = 318,423; middle row) and protein-coding regions (n = 262,025; bottom row). **c,** Nucleotide-resolution features of the 6 hepatocyte candidate enhancer core regions shown in bottom track of panel **a**. Tracks show basewise conservation (phyloP; 60 placental mammals) and model-derived saliency scores (evolution-aware model, CREsted: *crested.tl.contribution_scores*). **d,** Relationship between cell-class-specific enhancer prediction scores vs. cell-class-specific expression of nearby genes. We regrouped our mouse developmental scRNA-seq timelapse data^40^ into 32 cell classes, and generated normalized pseudobulk expression profiles for each. For each of ~22,000 protein-coding genes across 32 cell classes, cell-class-specific expression was defined as the log_2_ fold-change relative to the gene’s median expression across all cell classes. Each of the 318,423 cell-class-specific enhancer predictions of the evolution-aware model was linked to each gene within 100 kb, and such pairings assigned the corresponding cell-class-specific expression value. Enhancer-gene pairs were then jointly stratified by distance (*x*-axis; 5-kb bins with 2-kb sliding steps) and GPS (colors). Within each distance-score bin, the *y*-axis shows the mean cell-class-specific gene expression across all enhancer-gene pairs in that bin, after subtracting a background estimate obtained from 100 permutations in which enhancer-cell class assignments were randomly shuffled. Smoothed trends were fit using *geom_smooth* in ggplot2.

Although the evolution-aware model was trained on chromatin accessibility, we introduced modifications to isolate distal enhancer grammars (training on syntenically retained, evolutionarily coherent peaks; zeroing out TSS-adjacent regions) and to mitigate overprediction artifacts (tandem repeat randomization). A focused examination of ~100 kb encompassing the fetal hepatocyte marker genes *Alb* and *Afp* illustrates the impact of these adjustments (Fig. 5a). Observed chromatin accessibility is minimal in erythroid cells, while hepatocytes exhibit prominent peaks at promoters and candidate distal enhancers, together with accessibility throughout their highly transcribed gene bodies (Fig. 5a, top row-pair). The evolution-naive CREsted model recapitulates peaks at putative distal enhancers, but also peaks at both the *Alb* and *Afp* promoters in hepatocytes, as well as at regions inaccessible in both cell types, some of which, including the prominent peak in the erythroid track, are coincident with tandem repeats (Fig. 5a, second row-pair).

In contrast, the evolution-aware model yields a markedly simplified landscape at this locus: six discrete hepatocyte-specific peaks (Fig. 5a, third row-pair), trimmed to six 154–474 bp core elements (Fig. 5a, fourth row-pair; Supplementary Fig. 9d). Tandem-repeat-coincident predictions and the *Alb* promoter peak are eliminated. One candidate enhancer (#6), lying 12–166 bp upstream of the *Afp* TSS (Supplementary Fig. 10a), is predicted as hepatocyte-specific, raising the question of whether it represents a ‘very promoter proximal’ enhancer or a cell-type-specific promoter — a distinction we revisit in the next section .

Genome-wide, the 318,423 candidate developmental enhancers exhibit nucleotide constraint intermediate between background and coding regions (Fig. 5b). Within the six candidate hepatocyte enhancers at the *Alb-Afp* locus, high-saliency positions^2,67^ often coincide with bases exhibiting high phyloP scores^68,69^ and predicted binding sites for hepatocyte-related transcription factors (Fig. 5c). This pattern is also observed at the *Tcf20* and *Sox2* loci, which, in contrast with *Alb-Afp,* harbor a diversity of cell classes among their predicted enhancers (Supplementary Fig. 10b–c). Although high-saliency bases (> 0.1) within predicted developmental enhancers comprise only 1%, 3% and 3% of bases at the *Alb-Afp*, *Tcf20* and *Sox2* regions, they account for 8%, 20% and 7% of nucleotides with phyloP > 2, respectively, after excluding coding sequence.

To evaluate whether enhancers predicted by the evolution-aware model inform cell-class-specific gene expression, we matched our mouse developmental scRNA-seq annotations^40^ to the 32 cell classes and calculated cell class-specific expression vectors for ~22,000 protein-coding genes as their log_2_-fold change relative to the gene’s median across all cell classes. Candidate enhancers were binned by distance to each TSS and GPS. Aggregate plots show a clear dependence of gene expression on both predicted enhancer strength and proximity (Fig. 5d; Supplementary Fig. 10d), with both contributing independently within a given cell class. This result supports that evolution-aware model-predicted enhancers inform gene expression across major developmental lineages, and yields an aggregate estimate of distance-dependent decay in enhancer influence — albeit one confounded by genome architecture, as apparent decay with distance may partly reflect the increasing probability of intervening insulators or boundary elements. Notwithstanding this caveat, these results demonstrate that single-cell chromatin accessibility data, coupled with evolution-aware modeling, yields cell class-specific regulatory grammars that predict *in vivo* patterns of cell type-specific gene expression at the scale of the whole organism.

### Evolutionary augmentation enhances the robustness of cross-species inference

The evolution-aware model substantially improved mouse-genome-wide inference of distal regulatory grammars, but two key limitations remained. First, when we applied it in a cross-species manner, the resulting predictions were miscalibrated. For example, human orthologs of the 354,450 mouse windows scored in a correlated but attenuated manner (Supplementary Fig. 11a–b; Pearson’s *r* = 0.50; log_2_ fold-difference *Hs*/*Mm*: median −0.64, mean: −0.59). Further concerning, when we applied it to a 200-kb window centered on the *Afp* TSS across 136 mammalian genomes for which this region was contiguously assembled (chr5: 90,390,737–90,590,737, which encompasses the 100 kb region highlighted in Fig. 5a and an additional ~20 kb upstream and ~80 kb downstream; mean recoverable sequence: 191 ± 21 kb), the preponderance of hepatocyte-specific enhancer predictions that we observed for *Mus musculus* was largely restricted to other *Mus* species (Supplementary Fig. 11c). Regardless of the cause(s) (*e.g.* compositional differences; genuine evolutionary divergence), this miscalibration challenges our goal of evolutionary transfer learning.

Second, the effective training set was limited, with some cell classes represented by only around 1,000 enhancers — a paucity anticipated to worsen as we move from cell class (n = 36) to cell type (n = 140) resolution. Together, these observations highlight a core constraint: robust inference of mammalian *cis*-regulatory landscapes across hundreds to thousands of cell types likely requires a substantially larger and more diverse training corpus than any single genome can provide.

**Figure 6.**
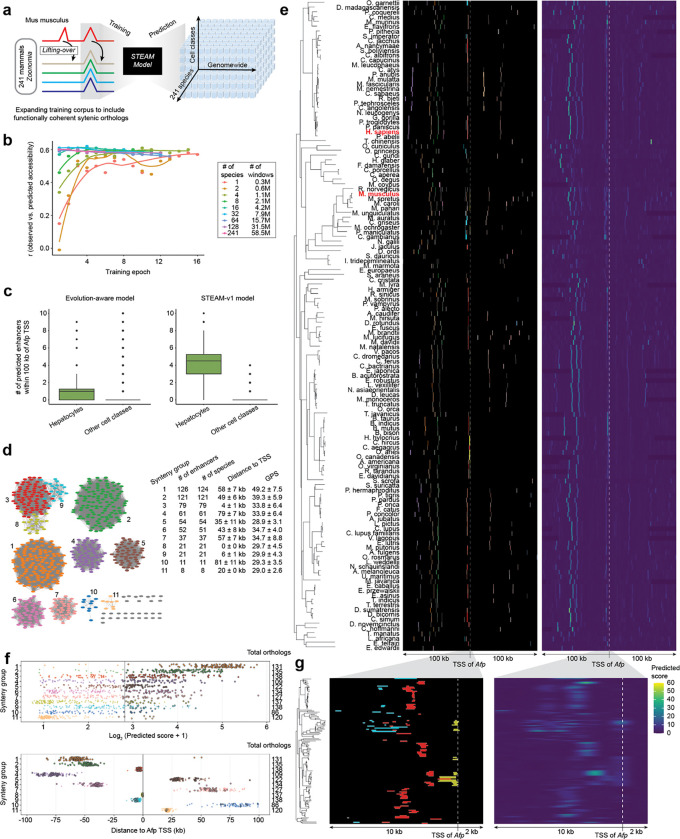
Evolutionary augmentation generalizes genomewide regulatory grammar inference across Mammalia. **a,** Evolutionarily coherent, syntenic orthologs of 354,450 × 100-bp mouse windows from 241 Zoonomia mammals^3^ were incorporated to train STEAM-v1, which was then applied to generate enhancer predictions spanning 32 cell classes for a 200 kb region centered on the *Afp* TSS in 136 species (panel **e**), as well as for whole-genome inference (human hg38/hg19, mouse mm10, and 239 additional Zoonomia genomes^3^). **b,** Models were trained on mouse windows and qualifying orthologs from progressively more species (1, 2, 4, …, 128, 241), expanding the corpus from 0.3M to 58.5M sequences. The 1-species model used mouse only; the 2-species model added human; subsequent models added randomly selected mammals. All models trained for up to 20 epochs with early stopping, except the 128- and 241-species models (manually stopped at epochs 12 and 6). Performance was evaluated at each checkpoint on a held-out mouse set of 22,014 sequences. **c,** The mouse *Afp* TSS was lifted over to 240 Zoonomia^3^ genomes and ±100 kb extracted; 136 species with ≥50 kb of contiguous recoverable sequence on each side were retained. The evolution-naive (left) and STEAM-v1 (right) models were applied to each syntenic locus (tiling, tandem repeat mitigation, GPS-based enhancer calling calibrated on mouse genome-wide scale, model-based trimming), and hepatocyte enhancer counts per species compared against the other 31 cell classes. Boxplot center lines: medians; box limits: 25th–75th percentiles. **d,** 621 hepatocyte enhancers predicted by STEAM-v1 across 136 species within 200 kb of the *Afp* TSS were lifted over between species; overlapping pairs were considered connected. The resulting graph forms 11 major synteny groups (clusters 1–11); remaining enhancers form smaller groups or singletons (grey). For each major synteny group: number of predicted enhancers, number of species represented, mean distance to *Afp* TSS, and mean GPS score are reported. **e,** Predicted hepatocyte chromatin accessibility from STEAM-v1 across 136 mammalian species within 200 kb of the *Afp* TSS. Left: phylogenetic tree for the 136 species. Middle: 621 hepatocyte enhancers colored by synteny group (as in panel **d**). Right: Predicted hepatocyte chromatin accessibility. **f,** For each of the 11 major synteny groups, orthologs in species lacking a called enhancer from that cluster were lifted over, merged, and treated as non-enhancer elements, yielding 591 enhancers and 786 non-enhancer elements. Top: log2-scaled predicted hepatocyte chromatin accessibility for enhancers (black-bordered) and non-enhancers, one row per synteny group. Bottom: distances to the *Afp* TSS for enhancers (black-bordered) and non-enhancers, one row per synteny group. In both plots, colors as in panel **d**. **g,** Subview of panel **e** spanning −10 kb to +2 kb of the *Afp* TSS across 136 species.

To overcome this, we sought to leverage the functional coherence of syntenic enhancers in a deeper way. Functionally constrained orthologous enhancers densely sample permissible sequence variation while preserving regulatory activity, and synteny enables their identification even in the face of substantial sequence divergence (Fig. 4e; Fig. 5b)^45,46^. We therefore expanded the training corpus to include syntenic orthologs of mouse-predicted enhancers from other mammals, increasing sequence diversity by ~200-fold while preserving cell class-specific labels (Fig. 6a) — analogous to the role of diverse MSAs in protein structure prediction.

Specifically, starting from the 354,450 mouse windows used to train the evolution-aware model, we incorporated orthologs from 240 additional mammals^3^ that satisfied: (i) syntenic retention (successful liftOver and expansion to 2,114 bp) and (ii) functional coherence (pairwise Pearson’s *r* ≥ 0.6 between evolution-naive prediction vectors; Fig. 4b,e). Orthologs were assigned the chromatin accessibility profiles of their corresponding mouse sequence, inevitably introducing label noise. However, we reasoned that the value of expanded sequence diversity would outweigh this noise — analogous to the expectation that a model trained to identify bicycles based on 20,000 low-resolution images taken will outperform a model trained on 100 high-resolution images, provided signal-to-noise remains sufficient.

We trained a series of ‘evolution-augmented’ models on the 10,000 most cell class-specific mouse windows and their qualifying orthologs, with increasing numbers of species (1, 2, 4, … 128, 241) expanding the corpus from 0.3M to 58.5M sequences (195-fold). The 1-species model was trained on mouse only, the 2-species model added human orthologs, and subsequent models added orthologs from additional randomly selected mammals. Models were trained for up to 20 epochs with early stopping based on validation performance, except for the 128- and 241-species models, which were manually stopped at epochs 12 and 6, respectively. Chromosome-based train/validation/test splits were shared between mouse and orthologs to prevent leakage, and mouse TSS-proximal regions were zeroed out as before, now together with their syntenic orthologs.

Performance on held-out mouse sequences improved with greater species inclusion but plateaued around 32 species (Fig. 6b). Notably, the primary impact of corpus expansion was to accelerate convergence rather than improve performance, possibly reflecting inherent limits to predicting accessibility from 2,114-bp windows alone (*e.g.* modulation of accessibility by epigenetic neighborhood, etc.). We selected the best-performing checkpoint on the validation dataset (32-species, epoch 4) and fine-tuned on the top 3,000 most cell-class-specific mouse windows and their orthologs, yielding the Synteny-aware Transfer learning for Enhancer Activity Modeling model (STEAM-v1).

As a first assessment of whether STEAM-v1 addressed the cross-species miscalibration of the evolution-aware model, we applied it to the 200-kb region centered on the *Afp* TSS across 136 mammalian genomes for which the evolution-aware model had failed to predict hepatocyte-specific enhancers beyond *Mus* (Supplementary Fig. 11c–d). For each syntenic locus, we performed predictions as previously described, including tiling, tandem repeat mitigation, GPS-based enhancer calling (calibrating on mouse genome-wide scale), and model-based trimming (Supplementary Fig. 9b,d).

The resulting differences are striking. While the evolution-aware model predicts 1.2 ± 1.7 hepatocyte enhancers per species (0.4 ± 1.5 for other cell classes), STEAM-v1 predicts 4.6 ± 1.7 (0.3 ± 0.5 for other cell classes) (Fig. 6c). As we have a strong expectation of hepatocyte-specificity at this locus, this corresponds to a 3-fold (evolution-aware) vs. 15-fold (STEAM-v1) signal-to-noise ratio. Moreover, the strong bias of the evolution-aware model for *Mus* is substantially mitigated with STEAM-v1 (mean predicted hepatocyte enhancers within *Mus* vs. all species: 8.2 vs. 1.2 (evolution-aware), 8.5 vs. 4.6 (STEAM-v1)).

Altogether, 621 hepatocyte enhancers in 136 species are predicted by STEAM-v1 at this locus (vs. 35 ± 19 for other cell types). However, 591 of these (95%) belong to 11 synteny groups, *i.e.* they have shared ancestry (Fig. 6d). To better understand these groups, we plotted their predicted accessibility scores and relative positions, together with those of an additional 570 orthologs from these same species and synteny groups that are not predicted as hepatocyte-specific enhancers (Fig. 6e–f). For some synteny groups, there is a clear separation of predicted-functional vs. predicted-non-functional orthologs that correlates with their phylogenetic distribution. For example, synteny group #11 contains only 8 predicted hepatocyte enhancers; these have STEAM prediction scores that are well separated from 102 non-functional syntenic orthologs and moreover all derive from old world monkeys (Fig. 6e–f). For other synteny groups, there is a more continuous distribution of predicted accessibilities that spans our threshold, with called enhancers more broadly distributed throughout the phylogeny (*e.g.* synteny group #5; Fig. 6e–f). As our threshold for calling enhancers is somewhat arbitrary, we cannot conclude with confidence whether elements falling just short of this threshold are nonfunctional.

Three synteny groups (#3, #8, #9) reside within 10 kb upstream of the *Afp* TSS (Fig. 6f). Upon closer inspection (Fig. 6g), synteny group #8 contains the aforedescribed *Afp* promoter-proximal enhancer (candidate enhancer #6; Supplementary Fig. 10a), centered 300 +/− 337 upstream of the *Afp* TSS across species. The hepatocyte GPS scores for this synteny group follow a long-tailed distribution across its 137 orthologs (median: 6.3; MAD: 8.8), with only 21 species (15%) exceeding the enhancer-calling threshold (median GPS among these: 26.7). Accordingly, candidate enhancer #6 in *Mus musculus* may be better classified as a ‘very proximal enhancer’ rather than a cell-type-specific promoter, as its hepatocyte specificity appears separable from the *Afp* TSS. This case also highlights the importance of distinguishing enhancer vs. promoter vs. other grammars, rather than solely focusing on accessibility as the target of inference.

### STEAM-v1 enables inference of human genome-wide enhancer landscapes

We applied STEAM-v1 to the human (hg38) and mouse (mm10) reference genomes at 100-bp tiling resolution to generate genome-wide distal enhancer prediction tracks across 32 cell classes, which collectively encompass all major lineages of the developing mammal (*HumMus-v1* tracks; Supplementary File 3, Supplementary File 4). Repeating the human-mouse ortholog comparison previously performed on the 354,450 evolution-aware windows (Supplementary Fig. 11a–b), we find that both correlation and attenuation are substantially improved with STEAM-v1 (Pearson’s *r* = 0.67; log_2_ fold-difference *Hs*/*Mm*: median −0.23, mean: −0.28; Supplementary Fig. 11e–f).

Across all cell classes, STEAM-v1 predicts 341,552 mouse enhancers (470 ± 288 bp) and 336,378 human enhancers (542 ± 310 bp); however, this close agreement is expected given our fixed-footprint peak-calling heuristic. In aggregate, these predictions cover 85 Mb and 90 Mb of the mouse and human genomes, respectively. Following liftover of mouse enhancer predictions to the human genome, 23% have a syntenic ortholog predicted as an enhancer for the same cell class, 49% have a syntenic ortholog that is not a matched prediction, 28% have no syntenic ortholog. Nearly identical proportions are obtained following liftover of human enhancer predictions to the mouse genome (24%, 49%, 27%).

**Figure 7.**
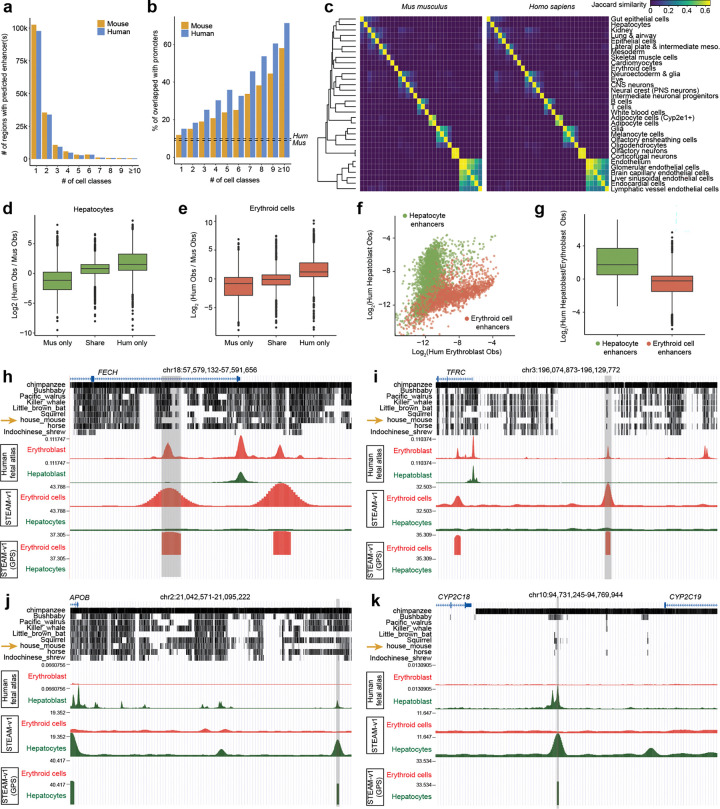
STEAM-v1 predicts human and mouse genome-wide enhancer landscapes. **a,** Genome-wide STEAM-v1 enhancer predictions were merged across cell classes, yielding 167,193 regions in mouse and 158,047 in human. Histogram of merged regions by the number of cell classes for which they are predicted enhancers, in mouse and human. **b,** For each category in panel **a**, the percentage of merged regions overlapping a promoter (± 2.5 kb around an annotated TSS) is shown for mouse and human. Background (dashed lines) was estimated from 10,000 randomly selected regions matched for size distribution. **c,** Jaccard similarity between all pairwise cell class combinations, calculated as the intersection over union of predicted STEAM-v1 enhancers, shown for mouse (left) and human (right). Merged groups composed of ≥7 cell classes (see panel **b**) were excluded from this analysis. The diagonal was set to the maximum non-diagonal value. Rows and columns for both species are ordered identically by hierarchical clustering on the mouse cell classes. **d,** Human and mouse predicted hepatocyte enhancers were reciprocally lifted over, and successful liftovers intersected to define three sets: mouse-only (n = 4,948), human-mouse shared (n = 2,597), and human-only (n = 5,790) predictions. Observed chromatin accessibility from human fetal liver hepatoblasts^51^ and mouse hepatocytes (this study) was extracted at averaged base resolution, and the distribution of log_2_-scaled human/mouse fold-changes is plotted for the three enhancer sets. Boxplot center lines: medians; box limits: 25th-75th percentiles. **e,** Same as panel **d** for erythroid enhancers: mouse-only (n = 3,492), human-mouse shared (n = 1,045), and human-only (n = 4,353) predicted enhancers with successful cross-species liftover. Observed chromatin accessibility from human fetal liver erythroblasts^51^ and mouse definitive erythroid cells (this study) was used. **f,** Human-specific hepatocyte (n = 3,239; green) and erythroid (n = 3,842; red) predicted enhancers failing liftover to the mouse genome are plotted by observed chromatin accessibility in human fetal liver erythroblasts (*x*-axis) vs. hepatoblasts (*y*-axis), log_2_-scaled^51^. **g,** For the same sets of human-specific enhancer predictions failing liftover to the mouse genome shown in panel **f**, the log_2_-scaled ratio of observed human hepatoblast to erythroblast accessibility is compared between hepatocyte-predicted (left) and erythroid-predicted (right) enhancers. Boxplot center lines: medians; box limits: 25th–75th percentiles. **h,** Genome browser view of a human erythroid-specific enhancer with no syntenic mouse ortholog, located in the first intron of *FECH*. Tracks show (top to bottom): Zoonomia 241-species alignment^3^, observed liver erythroblast and hepatoblast accessibility from the human fetal atlas^51^, STEAM-v1 predicted erythroid and hepatocyte accessibility, and GPS-scaled core enhancer predictions. Generated using the UCSC Genome Browser (hg38). **i,** Same as panel **h** for a human erythroid-specific enhancer with no syntenic mouse ortholog, located ~26 kb upstream of the *TFRC* TSS. **j,** Same as panel **h** for a human hepatocyte-specific enhancer with no syntenic mouse ortholog, located ~50 kb upstream of the *APOB* TSS. **k,** Same as panel **h** for a human hepatocyte-specific enhancer with no syntenic mouse ortholog, located ~15 kb upstream of the *CYP2C19* TSS, in the intergenic region between *CYP2C18* and *CYP2C19*.

Merging overlapping predictions yields 167,193 mouse (158,047 human) regions, of which 61% (62%) are predicted enhancers for a single cell class, 21% (21%) for two cell classes, and 17% (17%) for three or more cell classes (Fig. 7a). In both species, multi-class predictions are enriched for coincidence with TSSs (± 2.5 kb) (Fig. 7b), suggesting that the challenge of contaminating promoter grammar, although mitigated, may not be fully resolved. Remarkably, Jaccard-based co-occurrence analysis reveals a nearly identical structure for mouse and human enhancer predictions, with developmentally or functionally related lineages co-localizing most strongly (Fig. 7c).

For an initial assessment of human STEAM-v1 predictions, we turned to the Domcke *et al.* human fetal chromatin accessibility atlas, which we previously generated from midgestational organs via sci-ATAC-seq3^51^. As the Domcke *et al.* pseudobulk tracks were generated on hg19, we also applied STEAM-v1 to hg19 at 100-bp tiling resolution, yielding 336,745 human enhancer predictions (Supplementary File 5). We focused on erythroblast and hepatoblast accessibility profiles from fetal liver — the human counterparts of our mouse definitive erythroid and hepatocyte cell classes, respectively.

We first examined predicted human and mouse enhancers for these cell classes that have a syntenic ortholog in the other species — regardless of whether that ortholog was itself a predicted enhancer — as for these regions we have cell-class-matched observed accessibility data in both species. Reassuringly, predicted human-only enhancers were substantially more accessible than predicted mouse-only enhancers in the corresponding human cell class: an 8.8-fold difference for hepatocyte predictions, and an 8.5-fold difference for erythroid predictions (difference in geometric means; Fig. 7d–e) — consistent with STEAM-v1 correctly predicting species-specific regulatory differences at syntenically shared regions.

As a more stringent test, we examined predicted human enhancers that failed liftover to mouse entirely, *i.e.* regions for which there were neither human accessibility measurements nor even labeled mouse syntenic ortholog sequences available to STEAM-v1. As we also lack mouse accessibility measurements for these regions, we instead examined human hepatoblast/erythroblast accessibility ratios for both hepatocyte (n = 3,239) and erythroid (n = 3,842) predicted enhancers, and observe a mean 7.1-fold difference in the expected direction (difference in geometric means; Fig. 7f–g). Collectively, these results provide direct empirical validation of evolutionary transfer learning: STEAM-v1 infers human cell class-specific regulatory activity despite having been trained exclusively on mouse molecular data.

We highlight four examples of STEAM-v1 human-specific predicted enhancers with no mouse ortholog (Fig. 7h–k): (i) *FECH*: In the first intron of *FECH*, which encodes an erythroid-specific enzyme performing the final step of heme production, STEAM-v1 predicts a strong erythroid-specific enhancer (GPS 36.5), aligning precisely with an observed accessibility peak specific to human erythroblasts (Fig. 7h). Syntenic orthologs of this enhancer are intact in macaque, disrupted in other mammals, and absent in *Mus musculus.* (ii) *TFRC*: A similar case (erythroid GPS 36.1) resides ~26 kb upstream of *TFRC*, which encodes the erythroid-specific, iron-uptake-essential transferrin receptor, here within a broader region lacking mouse synteny (Fig. 7i). (iii) *APOB*: STEAM-v1 predicts a strong human hepatocyte enhancer (GPS 36.9) ~50 kb upstream of the TSS of *APOB*, a key determinant of circulating LDL-cholesterol levels (Fig. 7j). This element is syntenically retained in nearly all mammalian genomes, with the notable exception of *Mus musculus*. (iv) *CYP2C19*: This locus encodes an enzyme that metabolizes many commonly prescribed drugs, and like many cytochrome P450 genes, it lacks a 1:1 mouse ortholog. Around 15 kb upstream of its promoter, STEAM-v1 predicts a strong hepatocyte-specific enhancer (GPS 34.7) which may explain *CYP2C19’s* liver-specific expression (Fig. 7k). This is the only accessible peak, and the only STEAM-v1 predicted enhancer, in the ~20 kb region between *CYP2C18* and *CYP2C19*, for which full synteny appears restricted to a subset of primates.

## DISCUSSION

In this study, we demonstrate that evolutionary diversity can serve as a powerful supervisory signal for concurrently learning the multitude of gene regulatory grammars that manifest during mammalian development. By combining three key ingredients — (i) a time-resolved, whole-organism single-cell atlas of chromatin accessibility for a single species (reported here), (ii) a multi-output convolutional neural network framework (CREsted^2^), and (iii) hundreds of mammalian genomes with defined synteny blocks (Zoonomia^3^) — we achieved a modeling framework that learns the deeply conserved regulatory grammar of orthologous mammalian cell types. While our initial motivation was to infer genome-wide enhancer landscapes for human cell types that are inaccessible to direct profiling, an important outcome of this approach is that it enables enhancer prediction for 32 cell classes 241 mammalian genomes (7,721 genome-wide enhancer tracks).

A central insight underlying this work is that the effective training signal for generalizable regulatory models is limited by the diversity of sequences from which regulatory grammar can be inferred. The evolution-aware model demonstrated that filtering for evolutionary coherence improves inference of distal, cell type-specific regulatory programs. The key to scaling this approach is the ability to link orthologous regulatory elements across species, which we achieve through synteny. In mammals, synteny provides a particularly favorable regime: sequences are sufficiently diverged to richly sample the space of functional variation, yet sufficiently similar in genomic context to enable reliable alignment of even neutrally evolving sequences that are identical-by-descent^3,47^. This balance allows orthologous enhancers to be treated as diverse realizations of each cell type’s deeply constrained grammar — analogous to the role that multiple sequence alignments play in protein structure prediction. Our analyses suggest that although superficial measures of performance may plateau with the 241 mammalian genomes currently available, expanding this corpus further is likely to yield compounding benefits. In particular, this need will intensify as STEAM and any derivative strategies are extended to finer cellular resolution: whereas cell classes may be represented by thousands of enhancers per genome, more specialized grammars may be represented by far fewer. Additional genomes will be important for enabling reliable grammar inference as we progress from cell classes to the full granularity of mammalian cell type diversity — as well as to broader genomic scales, *e.g.* learning the regulatory logic governing entire loci rather than individual enhancers.

A key practical advantage of focusing on the mouse is that the entire organism is sufficiently small to be profiled as an intact whole — enabling a time-resolved, whole-animal cell atlas spanning early organogenesis through birth that captures essentially all major cell lineages as they emerge^39–43,70,71^. The growing availability of chromatin accessibility data provides an immediate opportunity to expand this approach, which may ultimately coalesce into a unified hierarchy of cell type-specific regulatory logic that broadly mirrors developmental lineage relationships. Indeed, apart from the 3.9 million single cell chromatin accessibility profiles reported here, other large-scale human or mouse scATAC-seq studies — of pre-implantation development, early embryonic development, extraembryonic tissues, adult tissues, brain regions, immune stimulation, etc. — sum to at least 3 million more cells, and more importantly include many cell type contexts that are not covered here^48,51,71–76^. Substantial progress can likely be made by extending the current framework to these datasets, as well as harmonized aggregations of scATAC-seq data^77^, an effort already underway.

Evolutionary transfer learning of regulatory grammars is not inherently limited to mammals. Among vertebrates, birds represent a particularly attractive target: hundreds of avian genomes have already been sequenced^78^, though time-resolved, single-cell molecular data are lacking. Teleost fishes such as zebrafish offer complementary advantages, including exceptionally rich time-series single-cell chromatin accessibility data^36,38^, but the density of high-quality sequenced fish genomes remains lower. Among and within these major taxa, one can anticipate a mix of deeply shared grammars and lineage-specific innovations. Looking further ahead, we envision frameworks that accommodate phylogenetic distance explicitly, allowing models to both constrain and permit divergence in regulatory logic as a function of evolutionary separation. Taken to its limit, sufficiently large collections of syntenic sequences may enable regulatory grammars to be parsed even in the absence of direct functional measurements, through unsupervised or weakly supervised approaches that exploit the tendency of syntenic sequences to preserve regulatory function. Our current framework represents a first step in this direction.

These advances also point toward new possibilities for human genetics, wherein progress in elucidating causal variants underlying tens-of-thousands of GWAS associations has been stalled by the difficulty of interpreting sequence variation across the full diversity of *in vivo* cellular states given the sharply limited number of contexts accessible to *in vitro* or *in vivo* functional experiments at scale^79^. Predictive models that capture the full breadth of *cis*-regulatory grammars have the potential to overcome these limitations, enabling *in silico* exploration of variant effects across myriad cell types and contexts. In this view, large-scale functional genomics datasets will serve not merely as end products, but as training substrates for increasingly generalizable models of context-aware and context-complete gene regulation, often by means of evolutionary transfer learning from model organisms.

In summary, we envision that thousands of cell type-specific regulatory grammars can be learned by extending this framework across mammalian and broader taxonomic diversity, and at multiple scales — from individual enhancers to entire regulatory loci. These models will initially be multi-class^2^ rather than single-track, and thus better positioned to predict gene expression, and regulatory variant effects, in a context-dependent manner. But ultimately they should be continuous rather than discrete, reflecting the fact that the unfolding of cell type identity through Waddington’s landscape is not a stepwise process but a seamless one^70^. Even as genomes have diversified, the fundamental structure of *trans*-acting regulatory programs — and the topography of Waddington’s landscape itself — remain deeply shared across placental mammals. By learning from the temporal unfolding of development across many species simultaneously, we can extract the consensus ontogeny of mammalian development, and then project it onto the human genome to predict human-specific aspects of this ontogeny — without ever requiring access to human embryonic, fetal or pediatric tissues.

There are important limitations to acknowledge, which we organize into two categories: methodological limitations of the current approach, and conceptual limitations that point toward future modeling directions.

Regarding methodological limitations: one potential concern is circularity. The evolutionary coherence filter used to curate training data relies on predictions from the evolution-naive model, which is itself trained on mouse chromatin accessibility data, raising the possibility that biases in the seed model propagate into training rather than being corrected. We note, however, that this circularity is not vicious in the logical sense — the filter is applied independently across hundreds of phylogenetically diverse mammalian genomes, and spurious predictions driven by mouse-specific sequence features are unlikely to be consistently reinforced across the full Zoonomia phylogeny. More importantly, the empirical improvements in cross-species calibration observed with STEAM-v1, including predictions at human-only enhancers that validate against human fetal chromatin accessibility data that the model did not have access to, provide evidence against systematic propagation of mouse-specific bias. Nevertheless, a more rigorous evaluation would involve cross-validation across both chromosomes and clades, and this remains an important direction for future methodological refinement.

A related concern is residual species bias. Although STEAM-v1 substantially reduced the Rodentia-restricted predictions of the evolution-aware model — as demonstrated at the *Afp* locus and by improved human-mouse cross-calibration — some bias likely remains, as evidenced by the persistent attenuation of human predictions relative to mouse (Supplementary Fig. 11e–f). The training corpus, while augmented with 240 mammalian genomes, is still anchored on mouse chromatin accessibility labels, meaning the model is ultimately supervised by a single-species molecular phenotype. Generating matched atlases in even a handful of phylogenetically strategic additional species would likely yield disproportionate improvements. More broadly, the predicted enhancer catalogs reported here should be treated as a first-generation resource: the synteny group analyses at the *Afp* locus illustrate that while STEAM-v1 correctly identifies well-conserved enhancers, some predictions remain ambiguous — potentially representing lineage-specific gains, subtle false positives, or elements falling just below our calling threshold. The synteny group framework offers a practical path for users to assess prediction robustness, and we expect the ratio of interpretable to ambiguous predictions to improve further as our understanding of how to assess confidence in individual enhancer predictions matures.

Regarding conceptual limitations: we wish to be transparent about our heuristic approach to suppressing promoter grammar. Zeroing chromatin accessibility within 2.5 kb of annotated TSSs during training is a blunt instrument, and we acknowledge that some readers may find it unsatisfying. It is not fully understood mechanistically why this step appears necessary — our working interpretation is that it forces the model to rely on sequence features beyond canonical promoter motifs, thereby biasing learning toward distal enhancer grammars. But we cannot rule out that it also discards genuine regulatory signals, particularly at loci where proximal enhancers and promoters are difficult to disentangle. The empirical evidence that this heuristic improves genome-wide inference is clear from our comparisons between the evolution-naive and evolution-aware models, but a principled, mechanistic replacement — ideally one grounded in an explicit model of enhancer vs. promoter grammar — remains an important goal. More broadly, developing models that cleanly separate enhancer, promoter, and other regulatory grammars during training, rather than relying on distance-based exclusion heuristics, is a key direction for the field. Finally, the modeling and computational implementation presented here was performed on a modest GPU cluster; more sophisticated architectures, larger input contexts, and scaled training may yield further performance improvements.

An obvious next step is to more deeply examine the 32 regulatory grammars learned by STEAM-v1. As a preliminary check, we examined motif enrichment among predicted cell-class-specific mouse and human enhancers, and found reassuring correspondence between the top enriched motifs and the key transcription factors associated with these lineages (Supplementary Figs. 12–13). A more comprehensive dissection using contemporary interpretability toolkits^80^ is underway.

To facilitate access to this work by the community, an interactive version of this preprint is at: is available at this link (ref ^4^), including the underlying count matrices, CREsted models, prediction tracks, code and reproducible figures. In addition to the STEAM-v1 genome-wide enhancer prediction sets for hg19, hg38 and mm10 (*HumMus* tracks), we have also applied the model to all Zoonomia genomes at 100 bp tiling resolution, for a total of 32 241 = 7,712 genome-wide enhancer tracks (*BabaGanoush* tracks). Peak calling, GPS calibration, and core element trimming for this larger set of mammalian genomes is underway, and will be released upon completion.

Our results suggest a renewed and modernized framing of the role of model organisms in the study of human biology. Model organisms remain the foundation of molecular biology: nearly everything we know about human development, physiology, and disease at the level of genes, molecules, and cells has its roots in insights first made in mice, flies, worms, fish, yeast, or bacteria. Yet this knowledge transfer has historically been ad hoc, and has not been systematically scaled to the era of AI and machine learning. Evolutionary transfer learning provides a framework for systematically leveraging the experimental richness of model organism data, together with the growing diversity of sequenced genomes, to make principled inferences about human biology. The current study employs the genome sequences of ~4% of extant mammalian species and a unimodal single-species molecular atlas spanning only half of mouse prenatal development. The headroom for improvement is therefore substantial. In this light, sequencing additional mammalian genomes and multimodal profiling the molecular, cellular, and developmental biology of key model organisms represent not merely exercises in cataloging biodiversity or satisfying scientific curiosity — though we wholeheartedly support both of those justifications in their own right — but critical investments in the data infrastructure needed to model human biology effectively. In short, Dobzhansky’s adage that nothing in biology makes sense except in the light of evolution is likely to be reinforced, not dampened, in the age of AI.

## METHODS

### Data reporting

For newly generated mouse data, no statistical methods were used to predetermine sample size. Embryo collection and sci-ATAC-seq3 data generation were performed by different researchers in different locations.

### Mouse embryos collection and staging

All animal use at The Jackson Laboratory was done in accordance with the Animal Welfare Act and the AVMA Guidelines on Euthanasia, in compliance with the ILAR Guide for Care and Use of Laboratory Animals, and with prior approval from The Jackson Laboratory animal care and use committee (ACUC) under protocol AUS20028.

The details of collecting 523 mouse embryos, ranging from embryonic day (E) 8.0 to P0, were described in a previous publication^40^. Briefly, C57BL/6NJ (strain #005304) mice were obtained from The Jackson Laboratory and maintained under standard husbandry conditions. Timed matings were set up in the afternoon, and vaginal plugs were checked the following morning. Noon on the day a plug was detected was designated as E0.5 and harvests were performed at intervals throughout the day or evening to ensure collection of samples within the desired 6 hour staging bins. At each developmental stage, individual decidua were removed and placed in ice cold PBS during harvest. Embryos were dissected free of maternal tissue and extraembryonic membranes, imaged, and then snap frozen in liquid nitrogen. Yolk sac tissue from each embryo was used to genotype for embryo sex , and all samples were stored at −80°C until further processing.

We employed a combination of staging approaches depending on the gestational age at harvest. To maximize temporal coherence, resolution, and accuracy, individual embryos were staged primarily on the basis of well-defined morphological criteria rather than gestational day alone (see ref ^40^ for full details). Briefly:

1. For E10.25 to E15.0, hindlimb bud morphology was used to estimate the absolute developmental stage of each sample via a combination of tools developed by Marco Musy in the Sharpe lab (embryonic Mouse Ontogenetic Staging System (eMOSS); https://limbstaging.embl.es/ and https://github.com/marcomusy/welsh_embryo_stager)^81,82^. Samples staged using eMOSS are denoted with “mE” to indicate morphometric embryonic day (*e.g.* mE13.5).

2. For E15.0 to E16.75, embryos were manually staged into bins based on changes in digit morphology as defined in ref ^40^.

3. Embryos from E17.0 to E18.75 were staged solely by gestational age.

4. P0 samples were harvested from litters at noon of the day of birth (parturition for C57BL/6NJ occurs between E18.75 and E19.0).

The images of individual embryos at each timepoint can be found in **Extended Data Fig. 1** of ref ^40^. Of note, only 36 samples from E10.0 to P0 were profiled using sci-ATAC-seq3 in this study.

#### Generating data using an optimized version of sci-ATAC-seq3

A total of six sci-ATAC-seq3 experiments were performed on 36 individual mouse embryos. At least one sample was included for every ~6 hour time interval from E10.0 to P0, except for the timepoint at E17.75. We tried to ensure that as many female mice as possible appear (29 females vs. 7 males) to avoid potential bias caused by sex differences, which we observed in a previous study^83^. A detailed summary of individual embryos can be found in Supplementary Table 1.

We generated the dataset using the sci-ATAC-seq3 protocol as described in a recent study^51^. The detailed protocol is also available at: https://www.protocols.io/view/sci-atac-seq3-ewov18xn7gr2/v1/v1. Briefly, snap-frozen tissues were pulverized and processed for nuclei fixation then banked for future experiments. On the day of experiment, frozen fixed nuclei were thawed, repermeabilized in Omni lysis buffer^84^, and diluted in ATAC-resuspension buffer (RSB: 10 mM Tris-HCl pH 7.4, 10 mM NaCl, 3 mM MgCl₂) supplemented with 0.1% Tween-20. For three-level indexing experiments at 384 × 384 × 384 resolution, 4.8 million nuclei (50,000 per well) were distributed across 96 wells. In this study, each experimental batch varied in the number of tissues per experiment to aim for at least 100,000 nuclei for early embryonic timepoint and increasing recovery for late developmental stage tissue. As per previous study, a mixture of GM12878 human and CH12-LX mouse cells were included as internal barnyard control.

For each tissue sample, nuclei were pelleted and resuspended in tagmentation master mix (Nextera TD buffer, 1X DPBS, 0.01% Digitonin, 0.1% Tween-20), aliquoted into wells (50,000 nuclei per well) of LoBind plates, and 2.5 μl Nextera v2 enzyme was added. Plates were incubated at 55°C for 30 min, and reaction was stopped by adding a stop solution (40 mM EDTA, 1 mM Spermidine) then incubated at 37°C for 15 min. Tagmented nuclei were pooled, pelleted, washed, and resuspended in ATAC-RSB with 0.1% Tween-20.

Phosphorylation master mix (1X PNK buffer, 1 mM rATP, T4 PNK) was added to tagmented nuclei, and distributed across four 96-well LoBind plates (12,500 nuclei per well) and then incubated at 37°C for 30 min. Ligation master mix (1X T7 ligase buffer, N5_splint, T7 DNA ligase) and N5 oligos (384 distinct barcodes) was added and incubated at 25°C for 1 hour, then stop solution (40 mM EDTA, 1 mM Spermidine) was added then incubated at 37°C for 15 min. Upon completion, reactions were pooled, pelleted, washed, and resuspended in N7 ligation master mix (1X T7 ligase buffer, N7_splint, T7 DNA ligase). Nuclei in ligation master mix was aliquoted across four 96-well LoBind plates, N7 oligos (384 distinct barcodes) added, and incubated at 25°C for 1 hour. Reactions were stopped and incubated at 37°C for 15 min.

Pooled nuclei were pelleted, resuspended in Qiagen EB buffer, and 1,000–4,000 nuclei were distributed across four 96-well plates. Cross-links were reversed by adding EB buffer, Proteinase K, and 1% SDS, then incubated at 65°C for 16 hours. Test PCR with SYBR green determined the optimal cycle number^1^, after which full amplification was performed using NEBNext HF 2x PCR Mastermix, BSA, and indexed P5/P7 oligos. Products were pooled, purified using Zymo Clean & Concentrate-5 and 1X AMPure beads to get rid of adapter dimers, and quantified on an Agilent 4200 Tapestation. Libraries were equimolar pooled at 2 nM and sequenced on an Illumina NextSeq 550 first and then sequenced deeply on a NovaSeq 6000 with custom primers and sequencing recipe (read 1: 51 cycles, read 2: 51 cycles, index 1: 10+15 dark+10 cycles, index 2: 10+15 dark+10 cycles). All splint and barcode sequences are provided in **table S7** of ref ^51^. The PCR duplication rate of individual samples ranges from 19% to 28% (Supplementary Table 2).

#### Processing of sci-ATAC-seq3 sequencing reads

Sequencing data was processed using the pipeline that we developed for sci-ATAC-seq3^51^ (https://github.com/shendurelab/sciatac_pipeline). Briefly, BCL files were converted to FASTQ format using Illumina’s bcl2fastq/v2.20. Each read was assigned a cell barcode composed of four components: the P5 end of the molecule included a row address for tagmentation and one for PCR; the P7 end of the molecule included a column address for tagmentation and one for PCR. To correct for the errors in these barcodes, we broke them into their 4 constituent parts and corrected them to the closest barcode within an edit distance of 2. If any of the four barcodes could not be corrected to a known barcode, the corresponding read pair was discarded. Reads with corrected barcodes contained in the read name were written out to a separate R1/R2 file for each of the samples in the dataset. Low-quality bases or adapter sequences were trimmed with Trimmomatic/v0.36^85^, and reads were mapped to the mm10 reference genome using Bowtie2^86^ with “-X 2000 −3 1” as options. Read pairs that did not map uniquely to autosomes or sex chromosomes, or had a mapping quality below 10, were filtered out using Samtools^87^. BAM files were then sorted, merged across sequencing lanes using Sambamba, and indexed for downstream analysis.

PCR duplicates within each cell were identified by determining the unique set of fragment endpoints. Within each sample, the resulting BED file of unique fragment endpoints for each cell was used for peak calling with MACS2^88^. Peak calls from all samples were then merged using bedtools to generate a master peak set (*n* = 376,574). Cell barcodes were distinguished from background using a modified approach from the 10x Genomics scATAC pipeline. A mixture of two negative binomials (noise vs. signal) was fitted to determine unique-read thresholds for calling cells in individual samples^51^, with a global cutoff of 500 unique reads per cell applied (Supplementary Table 2). Finally, sparse matrices counting unique reads within the master peak set were generated for each sample.

#### Initial cell-level QC and doublet removal

To identify high-quality cells, we applied the following criteria to each sample:

1. Unique reads: >1,000
2. Unique reads in peaks: 200 – 200,000
3. Fraction of reads in peaks: above a sample-specific cutoff (15 – 37%)
4. Fraction of reads in TSSs (+/−1 kb): above a sample-specific cutoff (8 – 13%)
5. Fraction of reads in ENCODE blacklist regions: 0.001 – 0.1
6. Nucleosome signal (calculated using the NucleosomeSignal function in Signac/1.9.0^89^): <10

After filtering, we merged the peak-by-cell sparse matrices from all 36 samples, which included six sci-ATAC-seq3 experiments across five NovaSeq runs, resulting in 4,344,905 cells. Dimensionality reduction was performed using Latent Semantic Indexing (LSI) followed by Principal Component Analysis (PCA). Peaks overlapping ENCODE blacklist regions, on sex chromosomes (to avoid batch effects between sexes), or present in <0.1% of cells were excluded. LSI was applied to the binarized peak-by-cell matrix by first weighting all peaks in each cell by log(total number of peaks accessible in that cell) (log-scaled “term frequency”), then multiplying these values by log(1 + the inverse frequency of each peak across all cells) (“inverse document frequency”). Singular value decomposition of the TF-IDF matrix produced a lower-dimensional PCA representation, retaining only dimensions 2–50, as the first dimension correlated strongly with read depth. The PCA matrix was then L2-normalized to account for differences in unique fragment counts per cell and used for downstream analyses. Most analyses were performed with BPCells/v0.1.0^52^. The L2-normalized PCA matrix was also used to perform Leiden clustering using sc.pp.neighbors(n_neighbors = 50) and sc.tl.leiden (resolution = 1) functions implemented in Scanpy/v1.6.0^90^, resulting in 27 cell clusters.

A limited number of doublet removal tools have been specifically developed for scATAC-seq (see: https://github.com/yuelaiwang/CEMBA_AMULET_Scrublet). To our knowledge, these tools fall into two main categories: 1) read count-based methods, such as AMULET^91^, and 2) algorithms adapted from scRNA-seq doublet detection, such as Scrublet^92^. We applied both AMULET and a modified Scrublet algorithm to identify potential doublets. Among the 27 cell clusters, two clusters (6 and 8) showed a high likelihood of being doublets, as identified by Scrublet or AMULET (Supplementary Fig. 1a). Cells with AMULET p-value < 0.05 or belonging to these two clusters were removed from downstream analysis, representing 9.4% of the total dataset.

Of note, we observed a reversal in the relative abundance of subnucleosomal fragments (<200 bp) versus mononucleosomal fragments (~200 bp) across timepoints and experiments. In early stages (before E16.0) or experiments 3–6, subnucleosomal fragments were more abundant than mononucleosomal fragments. In later stages (after E16.0) or experiments 7–8, this pattern was reversed (Supplementary Fig. 1b). This reversal was consistent across cell types, suggesting it may be driven by a technical factor.

#### Cell clustering and cell type annotations

After filtering low-quality cells and potential doublets, 3,937,903 cells from 36 embryos were retained, with a median of 3,922 reads and 828 peaks detected per cell. We repeated the filtering as described above, excluding peaks in ENCODE blacklist regions, on sex chromosomes, or present in <0.1% of cells, and applied LSI followed by PCA for dimensionality reduction. The L2-normalized PCA matrix (dimensions 2–50) was used for 3D Uniform Manifold Approximation and Projection (UMAP) embedding with n_components = 3, metric = “cosine”, n_neighbors = 50, and min_dist = 0.1. Seurat/v5^93^ was then used to compute a neighborhood graph (n_neighbors = 20) and Louvain clustering (resolution = 0.1), resulting in 29 major cell clusters.

Our recently generated scRNA-seq dataset^40^, from the same set of mice and spanning the same stages of mouse embryogenesis with comparable temporal resolution (~6 hours), technology (single-cell combinatorial indexing), and scale (11M nuclei RNA-seq vs. 4M nuclei ATAC-seq), is well annotated with >200 cell types, making it a valuable resource for multi-omics integration. We first matched timepoints between the two datasets and randomly selected 15,000 cells per timepoint from each dataset to reduce computational load. Dataset integration was performed using scGLUE/v0.3.2^50^ with default parameters, resulting in a 50-dimensional co-embedding space. Cell type labels were transferred from scRNA-seq to scATAC-seq using a kNN approach, with each scATAC-seq cell assigned the most frequent label among its 10 nearest scRNA-seq neighbors in the co-embedding space. Based on these labels, clusters in scATAC-seq were manually annotated and similar adjacent clusters were manually merged, resulting in 13 Level-1 cell lineages. In addition, we repeated dimensionality reduction using a new feature set of 545,114 genomic windows (5 kb) across the mouse genome. Cell clustering and annotation were robust to the choice of features, whether using merged peaks from each sample or tiled genomic windows (Supplementary Fig. 2d).

Next, for each Level-1 cell lineage, we repeated dimensionality reduction, clustering, and cell type annotation, slightly adjusting UMAP parameters (n_neighbors = 30, min_dist = 0.3), resulting in 36 Level-2 cell classes. The same procedure was then applied to each Level-2 cell class, resulting in 140 Level-3 cell types. The three hierarchical levels of cell type annotations are summarized in Supplementary Table 3. Of note, when annotating Level-2 cell class and Level-3 cell types, we re-performed integration between scATAC-seq and scRNA-seq, including only scRNA-seq cells expected to correspond to the same populations. For example, scRNA-seq cells from the major cell clusters of neuroectoderm & glia, intermediate neuronal progenitors, eye & other, CNS neurons, and neural crest (PNS neurons) were integrated with neuroectoderm cells in scATAC-seq.

These cell type labels are temporal and require further investigation. Therefore, we created a gene-by-cell matrix with gene-level accessibility scores, counting reads within gene bodies extended by 2 kb upstream. Using these gene-level accessibility scores, analogous to gene expression values, we manually verified whether cell-type-specific marker genes were specifically accessible in the corresponding cell types, revising a few annotations accordingly. The gene markers used for annotating each cell type are summarized in Supplementary Table 3.

#### Identification of inter-datasets correlated cell types using non-negative least-squares (NNLS) regression

To identify correlated major cell clusters between scRNA-seq and scATAC-seq datasets, we first calculated aggregate expression values (for scRNA-seq) or gene-level accessibility scores (for scATAC-seq) for each gene in each major cell cluster by summing the log-transformed normalized UMI counts of all cells in that cluster. For scATAC-seq, each Level-1 cell lineage was treated as a major cell cluster. To align with scATAC-seq, we manually grouped scRNA-seq cells into 13 major clusters—for example, consolidating neuroectoderm & glia, CNS neurons, neural crest (PNS neurons), intermediate neuronal progenitors, and eye & other into the neuroectoderm cluster, as shown in Fig. 2c.

Next, we applied non-negative least squares (*NNLS*) regression to predict gene expression in a target cell cluster (*T*_*a*_) in dataset A based on the gene expression of all cell clusters (*M*_*b*_) in dataset B: *T*_*a*_ = *β*_*0a*_ + *β*_*1a*_*M*_*b*_, based on the union of the 1,500 most highly expressed genes and 1,500 most highly specific genes in the target cell cluster. We then switched the roles of datasets A and B, *i.e.* predicting the gene expression of target cell cluster (*T*_*b*_) in dataset B from the gene expression of all cell clusters (*M*_*a*_) in dataset A: *T*_*b*_ = *β*_*0b*_ + *β*_*1b*_*M*_*a*_.

Finally, for each cell cluster *a* in dataset A and each cell cluster *b* in dataset B, we combined the two correlation coefficients: *β* = 2(*β*_*ab*_ + 0.01)(*β*_*ba*_ + 0.01) to obtain a statistic, where high values reflect reciprocal, specific predictivity. We repeated this NNLS analysis using Level-3 cell types, manually regrouping them into six major categories: erythroid, epithelial cells, endothelium, neuroectoderm, mesoderm, and white blood cells. Before analysis, scRNA-seq cell types corresponding to individual Level-3 scATAC-seq cell types were merged as needed, based on the matched cell types summarized in Supplementary Table 4.

#### Identification cell-type specific peaks

We reorganized fragments from each of the 140 Level-3 cell types and recalled peaks, merging them into a master list of 793,816 peaks. All subsequent analyses, including identification of cell-type-specific peaks, were based on this expanded peak set. Using the master peaks, we generated a new sparse peak-by-cell matrix by counting unique reads mapping to each peak in every cell, which was then binarized.

Cell-type-specific peaks were identified for each Level-2 cell class and each Level-3 cell type. To optimize computational efficiency and balance cell numbers across types, we randomly downsampled 10,000 cells per Level-2 cell class and 2,000 cells per Level-3 cell type. For each peak-cell type pair, a specificity score was calculated using Jensen-Shannon divergence, as previously described^48,51^.

1. Peaks overlapping ENCODE blacklist regions or exceeding two standard deviations from the mean log-scaled counts per peak were removed.
2. For each cell type, the median number of accessible sites per cell was calculated and log10-transformed. The scaling factor for each type was defined as the ratio of the average transformed value across all types to that type’s value.
3. For each peak, the proportion of accessible cells within each cell type was calculated. These proportions were normalized using the cell-type-specific scaling factors and then re-scaled across cell types within each peak to sum to 1.
4. For each peak and cell type, the re-scaled proportion was compared to a perfect distribution (1 for the target cell type, 0 for others) using Jensen-Shannon divergence. The specificity score was calculated as 1 minus the squared divergence.
5. The Jensen-Shannon specificity scores were squared and multiplied by the normalized proportion of accessible cells across cell types from step 3.

#### Overlap of cell-type-specific peaks with bulk tissue peaks in ENCODE mouse embryos

We first merged ATAC-seq peaks identified across timepoints (E11.5-E16.5) for each tissue in ENCODE mouse embryos^63^. Next, we randomly downsampled 10,000 cells per Level-2 cell class from our scATAC-seq dataset and removed peaks overlapping ENCODE blacklist regions. Specificity scores for each peak across level-2 cell classes were calculated using Jensen-Shannon divergence. For each level-2 cell class, the 2,000 most specific peaks were selected, and the number of base pairs overlapping bulk ATAC-seq peaks from ENCODE was counted and normalized by the total peak length of each tissue.

#### Evolution-naive modeling of chromatin accessibility across 36 Level-2 cell classes

We applied CREsted (https://github.com/aertslab/CREsted), a multi-output convolutional neural network (CNN) framework recently reported by the Stein Aerts lab^2^, to model Level-2 cell class-specific chromatin accessibility. For each of the 36 Level-2 cell classes, Tn5 cut site counts (approximated by fragment boundaries) were aggregated across all cells and normalized using CPM (counts per million; 10^6^ × raw count / total counts in the cell class). The resulting bedGraph files were converted to bigWig format using the bedGraphToBigWig tool. Fragments from each Level-2 cell class were also used to recall peaks, which were merged into a master peak list. Peaks located within ±2.5 kb of transcription start sites (TSSs) of both protein coding and long non-coding genes were excluded, resulting in a final set of 692,058 distal peaks. A peak by cell-class matrix with normalized counts was then used to identify cell-class-specific peaks by applying a softmax function to each peak. Model training was performed using the union of the top 10,000 peaks per cell class (299,281 peaks in total) together with bigWig signal tracks as input, primarily following default settings. Peaks from chromosomes 8 and 10 (~10% of all peaks) were used as the validation set, while peaks from chromosomes 9 and 18 (~10%) were held out as the test set. The remaining peaks (~80%) were used for training. Input sequences were recentered to 2,114 bp, and the top_k_percent parameter was set to 0.03 to normalize peak height across cell classes. Training used the peak_regression mode with the CosineMSELogLoss loss function, and all other configurations followed default settings (batch_size = 256, max_stochastic_shift = 3, learning_rate = 1e-3). The model was trained for up to 60 epochs with early stopping if no significant improvement was observed. After initial training, the model was fine tuned on a subset of 106,813 peaks comprising the top 3,000 most specific peaks per cell type, with the batch_size adjusted to 64 and the learning_rate set to 1e-4. The final model achieved an average Pearson correlation of 0.73 across the 36 Level-2 cell classes (range 0.63 to 0.82) on the held out test set, comparing predicted and observed accessibility within each cell type.

For genome-wide prediction in mouse, chromosomes and unlocalized sequences shorter than 2,200 bp were excluded. The remaining genome was segmented into 2,114 bp windows using a 100 bp sliding step, and segments containing fewer than 2,000 non-N bases were filtered out. The model trained on 36 Level-2 cell classes was then used for prediction, with predicted values assigned to the central 1,000 bp of each input sequence. Predicted values from overlapping sequences were averaged to generate the final chromatin accessibility profile at 100 bp resolution. A softmax function was applied to each 100 bp window to calculate cell class specificity, and the most specific windows for each cell class were selected using thresholds of Z-transformed predicted score ≥ 3 and Z-transformed specificity ≥ 3. Across the whole genome, 10% of 100 bp windows (2.79M) passed these thresholds for at least one cell class.

#### Evolution-aware modeling of distal regulatory grammars for 32 non-promoter clusters

We began with 2.79M 100 bp windows identified from the mouse genome that showed both high predicted accessibility and high predicted specificity (Z ≥ 3 for both metrics) in at least one cell class. As an initial filter, these windows were lifted from the mouse genome to 240 mammalian genomes from Zoonomia^3^ (https://zoonomiaproject.org/). We retained 1.47 million windows (53%) that were syntenically preserved in at least half of these mammals. As a second filter, for each retained window, we extracted 2,114 bp sequences centered on the syntenic position in each non mouse mammalian genome, resulting in a total of 296 million sequences (mean 201 orthologs per window). The CREsted model, trained only on mouse data, was applied to all sequences, and for each window we computed the Pearson correlation between the 36 dimensional predicted accessibility vector for the mouse sequence and those of its orthologs. The median correlation across orthologs for each window also showed a bimodal distribution. Using a cutoff of 0.6, we retained 547,317 windows that exhibited both strong evolutionary retention and coherent predicted regulatory activity across species.

To further organize these 547,317 windows, we constructed a kNN graph (k = 30) based on their 36 dimensional predicted accessibility vectors and applied Leiden clustering (resolution = 1). This analysis yielded 33 clusters, including one large cluster dominated by promoters. The remaining 32 clusters showed a strong one-to-one correspondence with our Level-2 cell classes and were annotated accordingly. A few exceptions were observed for cell classes that were not separable and were therefore merged (primitive and definitive erythroid; oligodendrocyte progenitors and committed oligodendrocyte precursors), or that were not distinguishable from the promoter dominated cluster and were therefore excluded (NMPs and erythroid niche cells).

A new model was trained on 354,450 windows from 32 non-promoter clusters (after filtering ENCODE blacklist regions; hereafter referred to as 32 cell classes). Model training followed a similar procedure as for the evolution-naive model. For each of the 32 non-promoter cell classes, normalized Tn5 cut site counts were extracted for individual candidate 100 bp windows, with regions within ±2.5 kb of TSSs of both protein coding and long non-coding genes masked. For cell classes derived from manually merged Level-2 cell classes (primitive and definitive erythroid; oligodendrocyte progenitors and committed oligodendrocyte precursors), the maximum value between the two merged classes was retained. A window by cell-class matrix with normalized counts was then used to identify class-specific windows by applying a softmax function to each window. Training was performed using the union of the top 10,000 windows per cell class (238,538 windows in total). Windows from chromosomes 8 and 10 were used as the validation set, while windows from chromosomes 9 and 18 were held out as the test set. The remaining windows were used for training. Input sequences were recentered to 2,114 bp, and the top_k_percent parameter was set to 0.03 to normalize window heights across classes. Training used peak_regression mode with the CosineMSELogLoss loss function, and all other settings followed defaults (batch_size = 256, max_stochastic_shift = 3, learning_rate = 1e-3). The model was trained for up to 60 epochs with early stopping if no significant improvement was observed. After initial training, the model was fine tuned on a subset of 95,139 windows comprising the top 3,000 most specific windows per cell class, with batch_size adjusted to 64 and learning_rate set to 1e-4.

We performed genome-wide prediction using the evolution-aware model. For this, as with genome-wide prediction using the evolution-naive model, the mouse genome was segmented into sliding 1,000 bp windows, and predictions averaged to 100 bp resolution. However, to mitigate false positive overpredictions induced by tandem repeats, we implemented a heuristic in the mouse reference genome (mm10) was modified by replacing tandem repeats, identified by Tandem Repeats Finder^64^ (TRF), with nearly random sequences that matched to local dinucleotide frequency (± 2,500 bp from the center position of each repeat). We repeated this procedure 10 times (*i.e.* genome-wide prediction on 10 modified versions of the mm10 mouse reference genome) and took the mean prediction for each window as its final prediction score.

#### Core region identification using evolution-aware predictions

After applying genome-wide prediction on the mouse genome using the evolution-aware mode, we ranked all genomic windows for each of the 32 non-promoter cell classes by their predicted accessibility. For each score, we defined *P* as the fraction of genomic windows with lower predicted scores and computed a Genome-wide Phred-like Score (GPS = −10 × log_10_[1 − P]). For each of the 32 cell classes, we selected 100-bp windows with a GPS score > 24.5 (n = ~100,000; ~10 Mb in aggregate) and merged adjacent windows. For each merged region, we extracted a 2,114-bp sequence (the CREsted model input length) centered on the region. We then iteratively replaced increasing amounts of sequence from the left and right ends with random DNA matched to local dinucleotide frequencies (± 2,500 bp from the center), following the pattern [20,0] → [20,20] → [40,20] → [40,40] → …, where each pair indicates the number of base pairs replaced at the left and right ends. At each step, both replaced sequence blocks were regenerated using newly sampled random DNA, and accessibility was re-predicted by the model. When the predicted score first fell below 90% of the original value, trimming from the end whose replacement block had most recently increased was halted, while trimming continued from the opposite end until it also caused the score to drop below the threshold. The core region was defined as the subsequence retained immediately before this final score decrease. Counting across all cell classes, a total of 318,423 core regions were identified, with an average length of 530 bp (± 316 bp, standard deviation). The score for each core region was defined as the predicted value immediately before dropping below 90% of its original level and was subsequently converted to a GPS score using the genome-wide predicted score distribution for the corresponding cell type.

#### Predicting cell-class-specific gene expression from enhancer activity

To identify the relationship between cell-class-specific enhancer prediction scores vs. cell-class-specific expression of nearby genes, we first regrouped our mouse developmental scRNA-seq timelapse data^40^ into 32 cell classes, and generated normalized pseudobulk expression profiles for each. For each of ~22,000 protein-coding genes across 32 cell classes, cell-class-specific expression was defined as the log_2_ fold-change relative to the median expression across all cell classes. Each of the 318,423 cell-class-specific candidate enhancers predicted by the evolution-aware model was linked to all nearby genes within 100 kb, and assigned the corresponding cell-class-specific expression value. Enhancer-gene pairs were then stratified jointly by genomic distance (5-kb bins with 2-kb sliding steps) and GPS scores. Within each distance–score bin, we computed the mean cell-class-specific gene expression across all enhancer-gene pairs, and subtracted a background estimate derived from 100 permutations in which enhancer-cell class assignments were randomly shuffled.

#### Evolution-augmented modeling enables cross-species inference

We started from the 354,450 × 100-bp mouse windows used to train the evolution-aware model, and incorporated their orthologs from 240 additional mammals^3^. Inclusion required: (i) syntenic retention (successful liftOver & expansion to 2,114 bp) and (ii) functional coherence (pairwise Pearson’s *r* ≥ 0.6 between 36-value evolution-naive prediction vectors for mouse vs. ortholog). Orthologs were assigned the same chromatin accessibility profiles across 32 cell classes as the corresponding mouse sequence. The evolution-augmented CREsted model was trained by: (i) initial training on the 10,000 most cell class-specific mouse windows and qualifying orthologs; (ii) chromosome-based train/validation/test splits shared between mouse (chromosomes 8 & 10: validation; 9 & 18: test; rest: training.) and orthologs, with each ortholog assigned to the same split as its corresponding mouse window, to prevent leakage; and (iii) zeroing regions within 2.5 kb of annotated mouse TSSs, and their orthologs. We trained models with increasing numbers of species (1, 2, 4, … 128, 241), which expanded the data corpus from 0.3M to 58.5M sequences (195-fold). The 1-species model was trained on mouse only, the 2-species model added human, and subsequent models randomly added additional mammalian genomes. All models were trained for up to 20 epochs with early stopping based on validation performance, except for the 128- and 241-species models, which were manually stopped early at epochs 12 and 6, respectively. We selected the model checkpoint exhibiting the highest performance on the validation set (32-species, 4 epochs) and fine-tuned it on the top 3,000 most cell-class-specific mouse windows and their orthologs. The final model, STEAM v1 (Synteny-aware Transfer learning for Enhancer Activity Modeling), was used to predict enhancer activity genome-wide across 241 mammals.

## Supplementary Material

Supplement 1 **Supplementary Table 1.** Summary of individual embryo samples.
**Supplementary Table 2.** Quality assessment of individual embryos after processing.
**Supplementary Table 3.** Three levels of iterative cell-type annotation, and the gene markers used for each Level-3 cell type.
**Supplementary Table 4.** Cell-type annotations from scATAC-seq and scRNA-seq show high concordance.
**Supplementary Table 5.** Genes showing significant differences in gene level accessibility scores between erythroid niche cells and primitive erythroid cells (*p* < 0.01).

Supplement 2

## Data Availability

An interactive version of this preprint, together with count matrices, CREsted models, prediction tracks, code and reproducible figures, is available is available at this link (ref ^4^). Code and scripts are available at https://github.com/ChengxiangQiu/jax-atac-code. The data generated in this study can be downloaded in raw and processed forms from the NCBI Gene Expression Omnibus under accession number GSE325776.
