# Supplementary material for: Evolutionary transfer learning enables organism-wide inference of mammalian enhancer landscapes
Source: bioRxiv. 2026 May 27:2026.04.07.717039. Originally published 2026 Apr 8. Preprint. [Version 2] doi: 10.64898/2026.04.07.717039 (PMC13081831; doi:10.64898/2026.04.07.717039)
Supplement: Supplement 2 [file NIHPP2026.04.07.717039v2-supplement-2.pdf]

bioRxiv preprint doi: https://doi.org/10.64898/2026.04.07.717039; this version posted May 27, 2026. The copyright holder for this preprint (which was not certified by peer review) is the author/funder, who has granted bioRxiv a license to display the preprint in perpetuity. It is made available under a CC-BY 4.0 International license.

SUPPLEMENTARY FIGURES

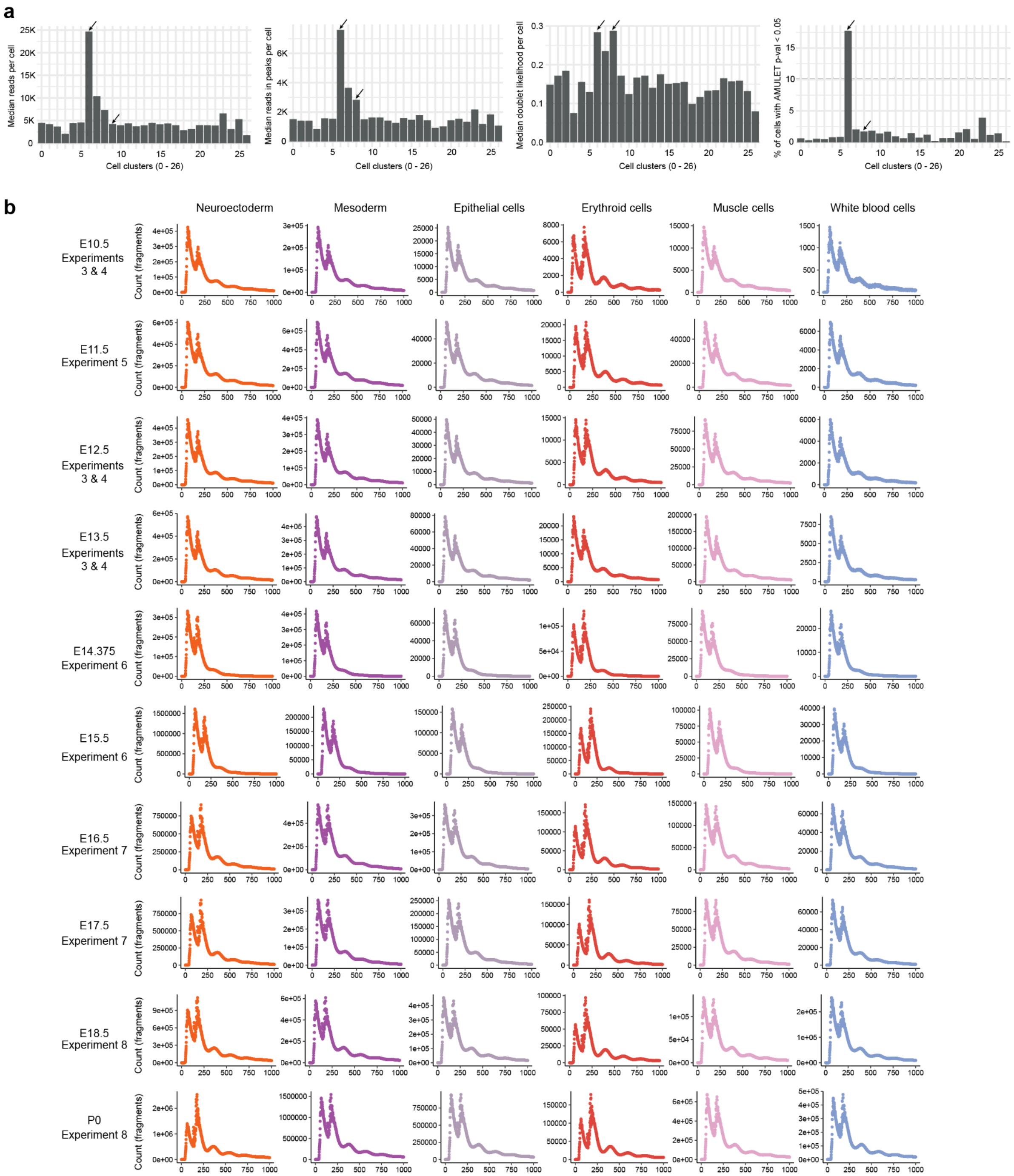

Supplementary Figure 1. Doublet detection and fragment size distributions.

a, Prior to doublet removal, we identified 27 cell clusters from 4.3 million cells using LSI-based dimensionality reduction and Leiden clustering with a resolution of one[94]. Doublet detection was then performed with both AMULET[95] or Scrublet[96]. From left to right: The median number of reads per cell, the median number of reads in peaks per cell, the median doublet likelihood calculated by Scrublet per cell, and the proportion of doublet cells detected by AMULET, are shown plotted for each of the 27 cell clusters. Cells from clusters 6 and 8 (highlighted by arrows in each plot) were identified as doublets and excluded from downstream analyses.

b, Distribution of fragment sizes varied across timepoints and experiments. Histograms of fragment sizes for cells from 10 representative timepoints (rows; one per day and at least one from each of the 6 experiments) for the six most abundant Level-1 cell lineages (columns) are shown. Fragments greater than 1 kb in length were discarded.

bioRxiv preprint doi: https://doi.org/10.64898/2026.04.07.717039; this version posted May 27, 2026. The copyright holder for this preprint (which was not certified by peer review) is the author/funder, who has granted bioRxiv a license to display the preprint in perpetuity. It is made available under a CC-BY 4.0 International license.

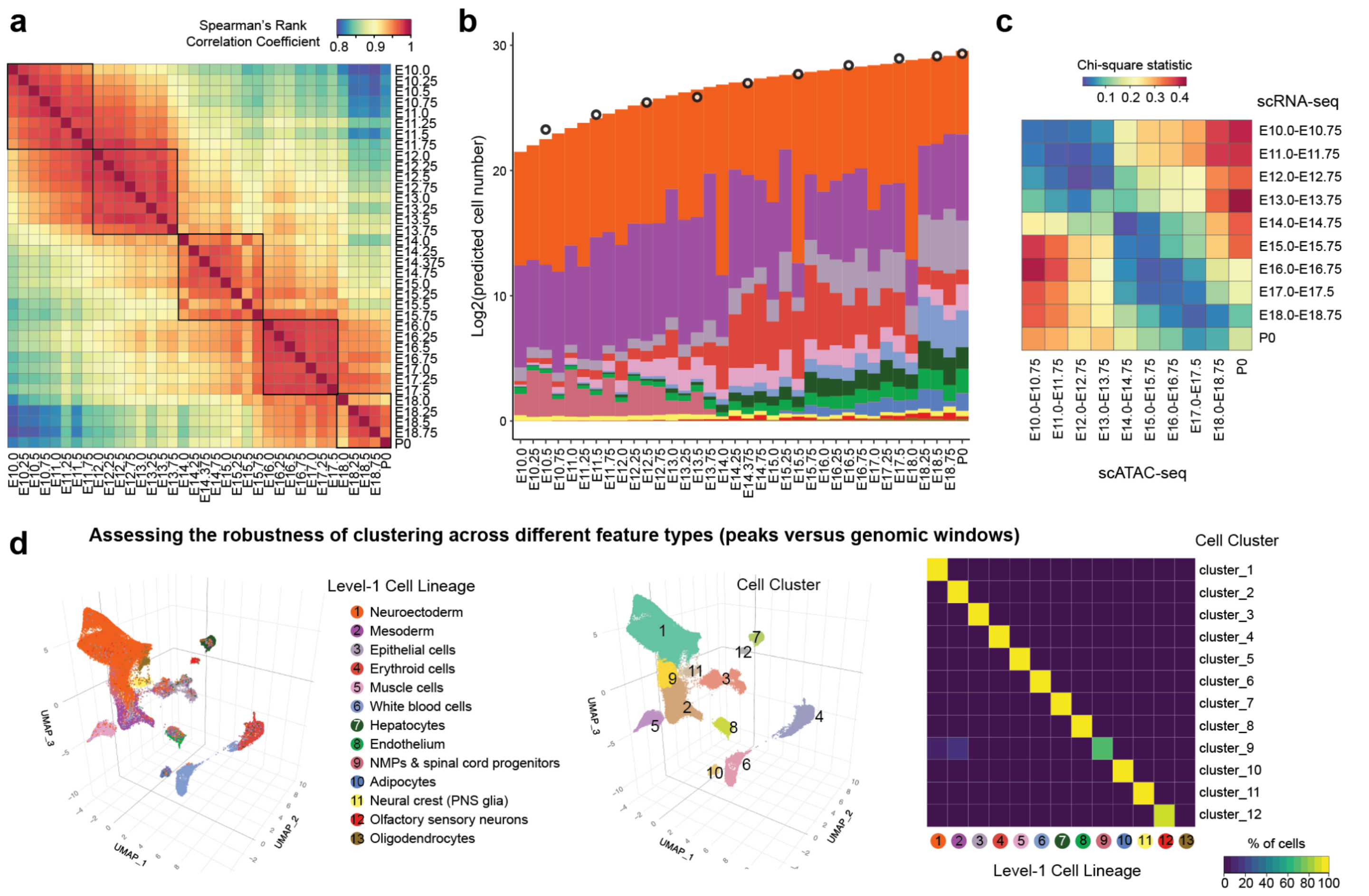

Supplementary Figure 2. Temporal dynamics and robustness of Level-1 cell lineage annotations.

a, Spearman correlations were calculated for pairwise combinations of 36 temporal bins, each generated by aggregating scATAC-seq data from a staged embryo after downsampling to a uniform number of cells per sample. Black rectangles are manually added and correspond to two-day intervals.

b, Composition of embryos from each 6-hour temporal bin by Level-1 cell lineage. The *y*-axis is scaled (log_2) to the estimated total number of cells in the embryo at each timepoint (see Qiu et al.[42] for details). Briefly, we isolated and quantified total genomic DNA from whole embryos to estimate total cell number at each of 12 stages spanning E8.5 to P0 (1-day bins, highlighted by black circles), and then applied polynomial regression to predict the log_2 scaled cell number at each of the 36 timepoints (treating P0 as E19.5 in the regression)[42].

c, Compositions across 12 Level-1 cell lineages were compared between scRNA-seq and scATAC-seq using the Chi-squared statistic, where higher values indicate greater differences. For this analysis, cells were grouped in four-day intervals. The NMPs & spinal cord progenitors and neuroectoderm Level-1 scATAC-seq annotations were merged for consistency with scRNA-seq annotations at this level of resolution, while major trajectories from scRNA-seq annotations[42] were merged as appropriate for consistency with the Level-1 scATAC-seq annotations as well.

d, To verify the robustness of our clustering strategy to feature choice (peaks vs. genomic windows), we re-performed dimensionality reduction on the read count matrix using 545,114 *x* 5 kb windows of the mouse genome as features. The resulting UMAP is shown twice, with cells colored by Level-1 cell lineage annotations (left) or the major cell clusters identified by the new features (middle). Adjacent major cell clusters were manually merged to achieve a similar resolution. The heatmap (right) shows the percentage of cells from each Level-1 cell lineage annotation (n = 13) within each major cluster (n = 12). Of note, 94% of oligodendrocytes (last column) were included in cluster 1, which predominantly overlaps with neuroectoderm.

bioRxiv preprint doi: https://doi.org/10.64898/2026.04.07.717039; this version posted May 27, 2026. The copyright holder for this preprint (which was not certified by peer review) is the author/funder, who has granted bioRxiv a license to display the preprint in perpetuity. It is made available under a CC-BY 4.0 International license.

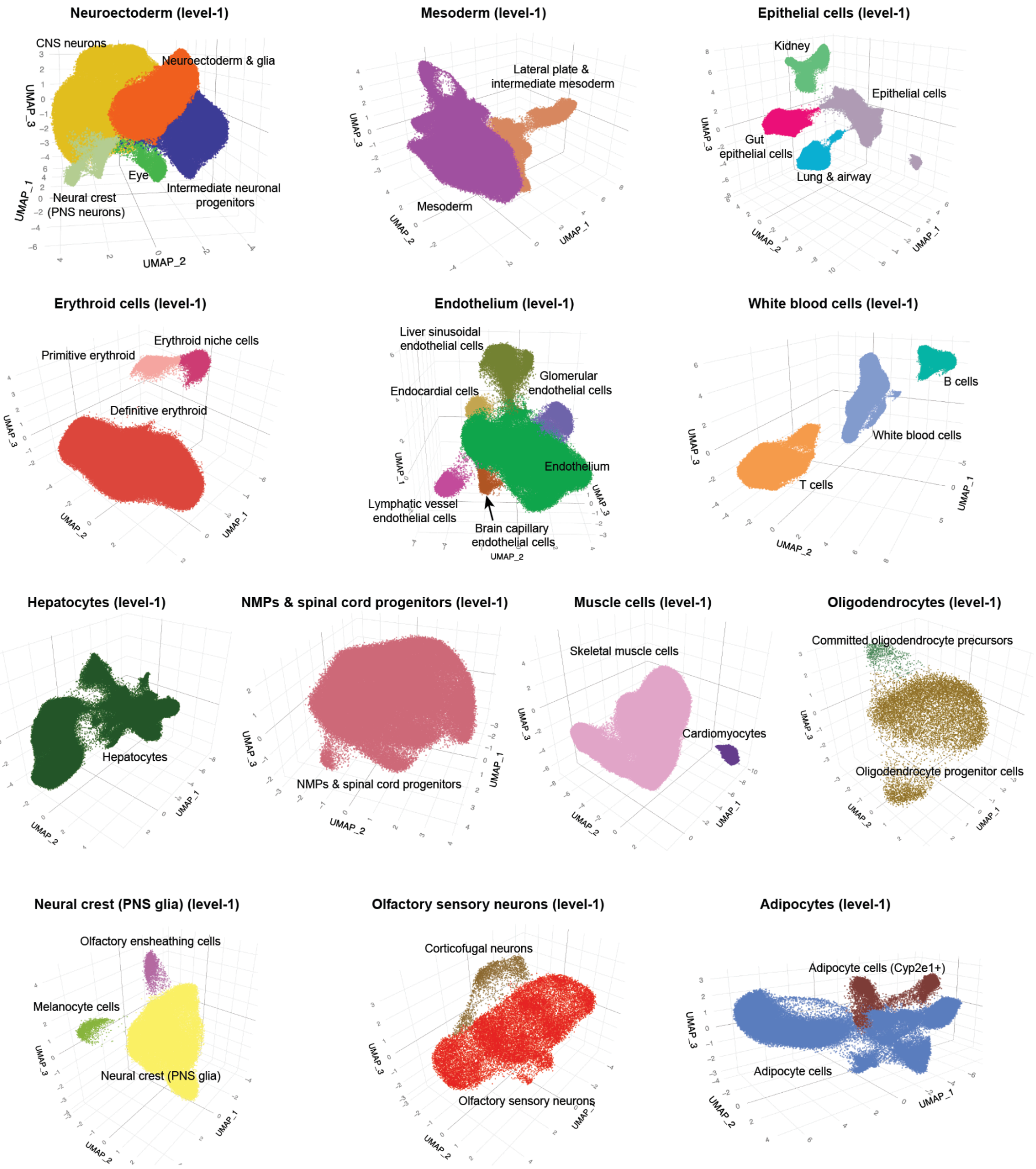

Supplementary Figure 3. Annotation of 36 Level-2 cell classes.

For each of the 13 Level-1 cell lineages, we performed subclustering and annotated the resulting 36 Level-2 cell classes using label transfer from our scRNA-seq atlas[42] along with at least two literature-supported marker genes per cell type label (**Supplementary Table 3**).

bioRxiv preprint doi: https://doi.org/10.64898/2026.04.07.717039; this version posted May 27, 2026. The copyright holder for this preprint (which was not certified by peer review) is the author/funder, who has granted bioRxiv a license to display the preprint in perpetuity. It is made available under a CC-BY 4.0 International license.

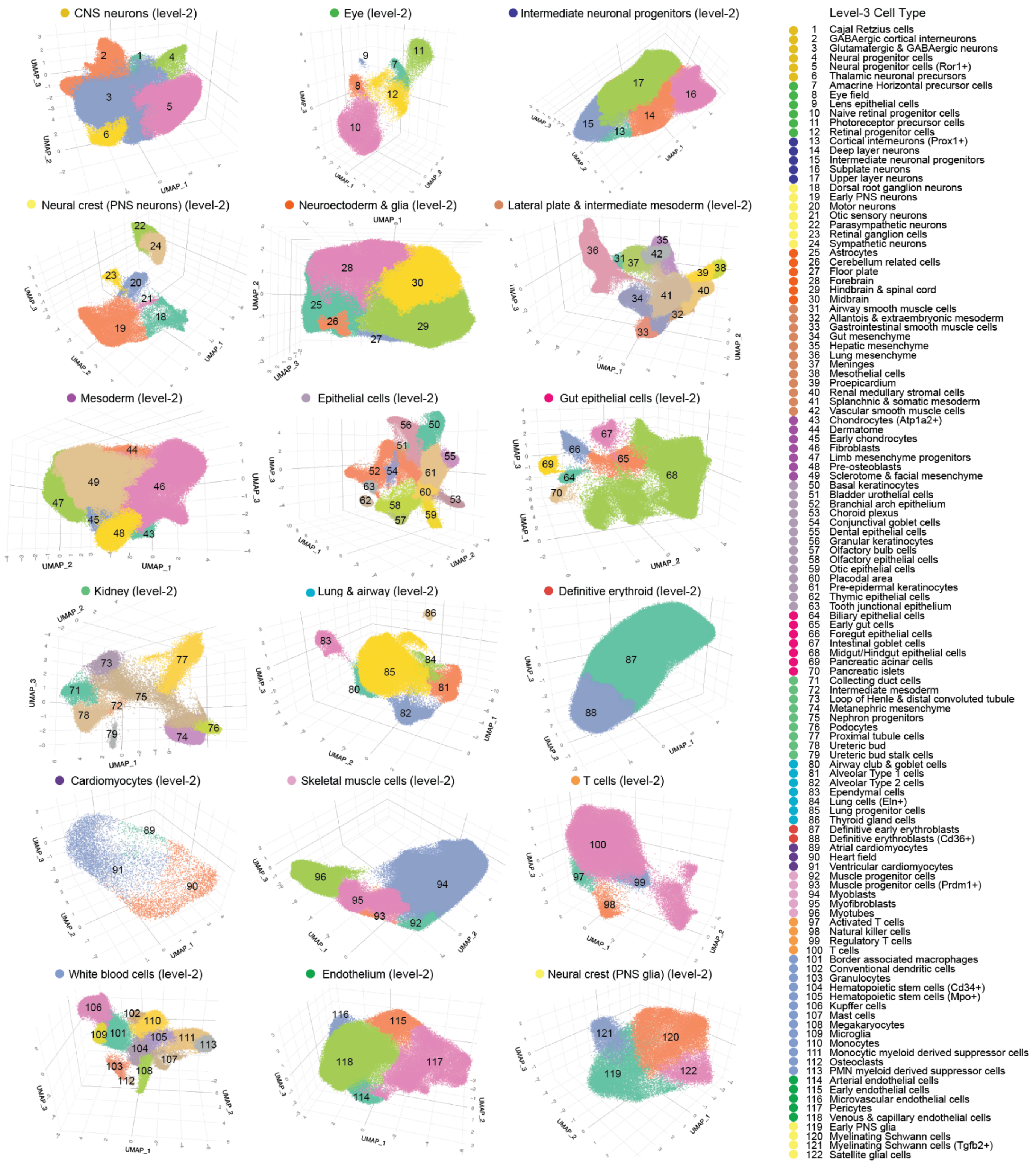

Supplementary Figure 4. Annotation of 140 Level-3 cell types.

For 18 of 36 Level-2 cell classes, we performed subclustering and annotated the resulting 122 Level-3 cell types using label transfer from our scRNA-seq atlas[42] along with at least two literature-supported marker genes per cell type label (**Supplementary Table 3**). The remaining 18 Level-2 cell classes were not subclustered, and are also represented among Level-3 annotations as cell types.

bioRxiv preprint doi: https://doi.org/10.64898/2026.04.07.717039; this version posted May 27, 2026. The copyright holder for this preprint (which was not certified by peer review) is the author/funder, who has granted bioRxiv a license to display the preprint in perpetuity. It is made available under a CC-BY 4.0 International license.

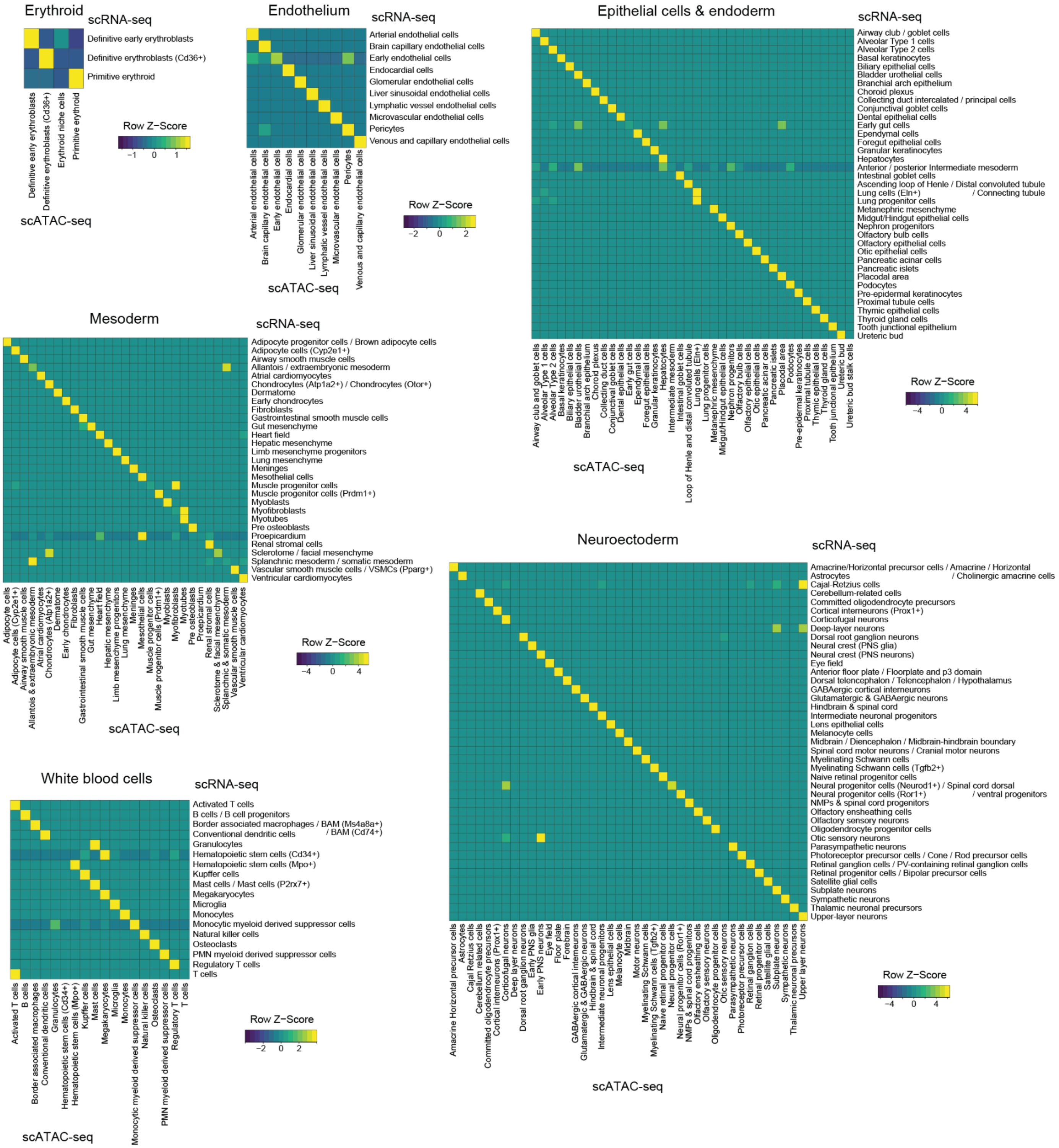

Supplementary Figure 5. Correlations between scRNA-seq and scATAC-seq cell type annotations based on non-negative least-squares (NNLS) regression.

After manually splitting 140 scATAC-seq Level-3 cell type annotations and 170 scRNA-seq individual or grouped cell type annotations[42] into six major developmental groups, we performed NNLS regression. The resulting heatmaps show the combined regression coefficients (NNLS; row-scaled) for scRNA-seq cell types (rows) vs. 13 scATAC-seq cell types (columns), within each group. Gene-level accessibility scores were used for scATAC-seq (see **Methods**). Prior to this analysis, some subsets of the 56 scRNA-seq cell types were merged prior to performing this analysis, as summarized in **Supplementary Table 4**. CNS: central nervous system. PNS: peripheral nervous system. NMPs: Neuromesodermal progenitors.

bioRxiv preprint doi: https://doi.org/10.64898/2026.04.07.717039; this version posted May 27, 2026. The copyright holder for this preprint (which was not certified by peer review) is the author/funder, who has granted bioRxiv a license to display the preprint in perpetuity. It is made available under a CC-BY 4.0 International license.

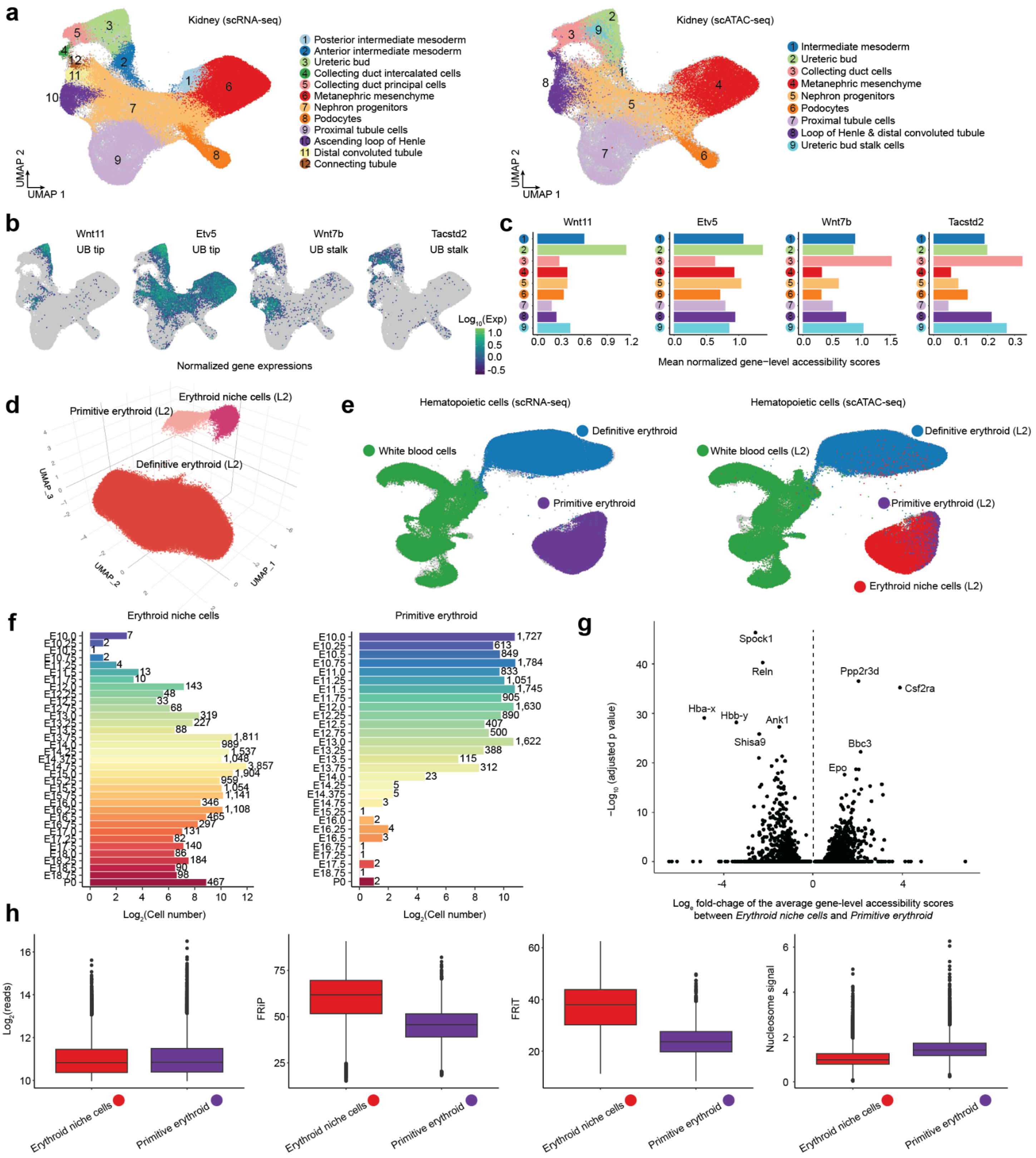

Supplementary Figure 6. Ureteric bud stalk cells and erythroid progenitors identified uniquely by scATAC-seq.

a, 2D UMAP visualization of 132,769 co-embedded cells corresponding to renal development, from published scRNA-seq[42] and scATAC-seq (this study) data, after integration with scGLUE[52]. The same UMAP is shown twice, highlighting cells from either scRNA-seq (left) or scATAC-seq (right) data.

bioRxiv preprint doi: https://doi.org/10.64898/2026.04.07.717039; this version posted May 27, 2026. The copyright holder for this preprint (which was not certified by peer review) is the author/funder, who has granted bioRxiv a license to display the preprint in perpetuity. It is made available under a CC-BY 4.0 International license.

b, The same UMAP as in panel **a**, colored by expression of marker genes which appear specific to the ureteric bud tip (*Wnt11*+, *Etv5*+) or stalk (*Wnt7b*+, *Tacstd2*+)[99].

c, In the scATAC-seq data, gene level accessibility scores were calculated by counting reads overlapping gene bodies plus 200 kb upstream, and those scores were normalized by the total score per cell and multiplied by 10,000. Mean accessibility scores across cells within each renal cell type were plotted for marker genes specific to the ureteric bud tip (*Wnt11*+, *Etv5*+) or stalk (*Wnt7b*+, *Tacstd2*+)[99].

d, 3D UMAP visualization of 336,656 scATAC-seq profiles from the erythroid cell lineage, colored by Level-2 cell class.

e, 2D UMAP visualization of 284,183 co-embedded cells corresponding to hematopoietic development, from published scRNA-seq[42] and scATAC-seq (this study) data, after integration with scGLUE[52], colored by Level-2 cell class. Included are all erythroid cell classes and the white blood cell cell class, but not B or T cell classes. The same UMAP is shown twice, highlighting cells from either scRNA-seq (left) or scATAC-seq (right) data.

f, The number ($\log_2$ scaled) of scATAC-seq profiles annotated as erythroid niche (left) and primitive erythroid (right) cells, sorted by staging bin.

g, In the scATAC-seq data, gene level accessibility scores for 21,960 protein-coding genes were compared between the erythroid niche and primitive erythroid annotations after randomly downsampling each group to 1,000 cells, using the *FindMarkers* function implemented in Seurat/v5[97]. The fold-change (natural log scaled) of the average scores between the two groups (*x*-axis) and the negative $\log_{10}$ adjusted p-value (*y*-axis) were plotted, with selected significant genes labeled. Full results are provided in **Supplementary Table 5**.

h, The number of unique reads, the percentage of reads in peaks (FRiP), the percentage of reads falling within TSSs (±1 kb) (FRiT), and the nucleosome signal, were compared between erythroid niche (n = 18,759) and primitive erythroid cells (n = 15,424). The nucleosome signal represents the approximate ratio of mononucleosomal to nucleosome-free fragments[93]. In the boxplots, center lines indicate the medians, and box limits denote the 25th and 75th percentiles.

bioRxiv preprint doi: https://doi.org/10.64898/2026.04.07.717039; this version posted May 27, 2026. The copyright holder for this preprint (which was not certified by peer review) is the author/funder, who has granted bioRxiv a license to display the preprint in perpetuity. It is made available under a CC-BY 4.0 International license.

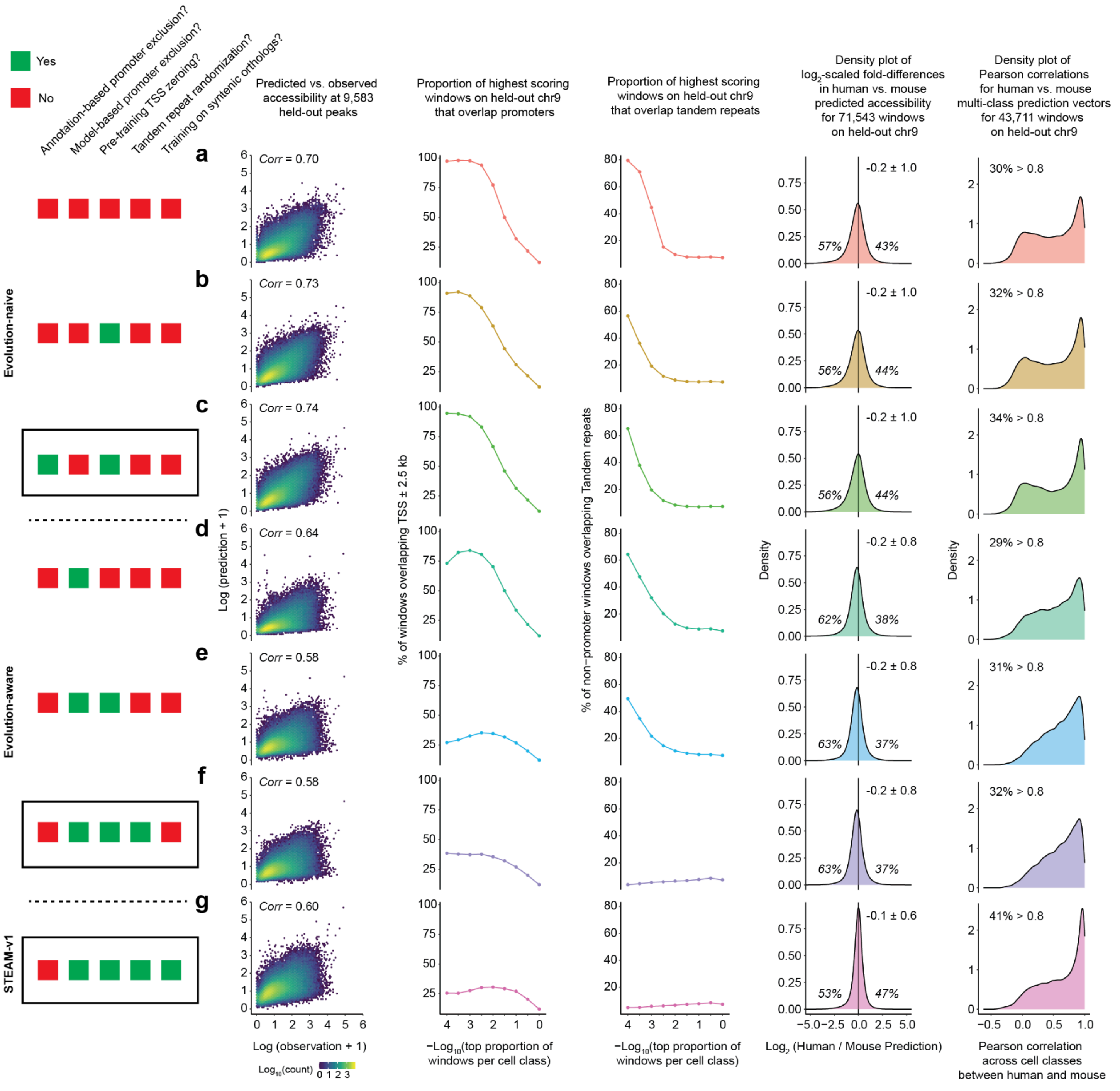

Supplementary Figure 7. Evaluating successive model iterations against the four modeling objectives.

Each row (panels **a-g**) corresponds to a model configuration. Each column corresponds to a metric chosen to evaluate one of the four objectives articulated in the main text. Rows are grouped by model iteration. Panels **a-c** correspond to several versions of the **evolution-naive** model, trained on 795,998 peaks obtained by merging peaks called on each of the 36 Level-2 cell classes. Panels **d-f** correspond to several versions of the **evolution-aware** model, trained on 354,450 100-bp windows that are syntenically retained, evolutionarily coherent, and excluded from the promoter cluster. Panel **g** corresponds to the **evolution-augmented** model (STEAM-v1), which extends the evolution-aware model by incorporating syntenic orthologs from additional Zoonomia mammals alongside the same 354,450 windows. Within each row group, panels vary along five modifications introduced to address specific failure modes: (i) annotation-based promoter exclusion (TSS ± 2.5 kb); (ii) model-based promoter exclusion (windows assigned to the promoter cluster as shown in **Fig. 4d**); (iii) manual zeroing of TSS ± 2.5 kb regions prior to training; (iv) tandem repeat replacement during genome-wide prediction

bioRxiv preprint doi: https://doi.org/10.64898/2026.04.07.717039; this version posted May 27, 2026. The copyright holder for this preprint (which was not certified by peer review) is the author/funder, who has granted bioRxiv a license to display the preprint in perpetuity. It is made available under a CC-BY 4.0 International license.

(**Supplementary Fig. 10b**); and (v) inclusion of syntenic orthologs from additional species. For each panel, green rectangles indicate a given modification was applied; red indicates it was not. The three models highlighted with black rectangles (panels **c**, **f**, **g**) correspond to the primary evolution-naive, evolution-aware and evolution-augmented models referenced throughout the manuscript.

The five columns evaluate the four modeling objectives as follows. **First column (Goal #1, prediction on held-out peaks):** Scatterplots of observed (*x*-axis; normalized Tn5 cut-site counts, log-n scaled) vs. predicted (*y*-axis; log-n scaled) chromatin accessibility across peaks held out during fine-tuning (**Fig. 3b**), shown for all 36 Level-2 cell classes (evolution-naive) or 32 cell classes (evolution-aware, STEAM-v1), colored by $\log_{10}$ scaled density. **Second column (Goal #2, learning distal enhancer rather than promoter grammar):** Predictions across 1.25M × 100 bp windows spanning mouse chr. 9 (**Fig. 3g**). For the top proportion of windows per cell class (*x*-axis, negative $\log_{10}$ scaled), the *y*-axis shows the percentage overlapping with TSS ± 2.5 kb. A model that has learned distal enhancer rather than promoter grammar should show low promoter overlap among its top-scoring windows. **Third column (Goal #3, avoiding overprediction at tandem repeats):** Same prediction outputs as the second column. For the top proportion of windows per cell class, the *y*-axis shows the percentage overlapping with tandem repeats among those not overlapping with TSS ± 2.5 kb. **Fourth and fifth columns (Goal #4, cross-species generalization):** Both evaluate a fixed set of 71,543 100-bp windows on mouse chr. 9 (selected using the evolution-naive model's prediction and specificity z ≥ 3) with successful liftOver to the human genome. The fourth column shows the density of $\log_2$-scaled fold-differences in predicted chromatin accessibility between human and mouse, with the percentages of windows with $\log_2$ fold differences > 0 and < 0, along with the mean and standard deviation, reported. The fifth column further filters this set to the 43,711 windows overlapping with mouse core enhancers from STEAM-v1 predictions, and plots the density of Pearson correlation across 32 cell classes between mouse and human predictions; the percentage of windows with Pearson correlation > 0.8 is reported.

a, Evolution-naive CREsted model trained on 306,736 windows (10,000 most specific peaks per class), followed by fine-tuning on 105,671 windows (3,000 most specific peaks per class), to predict chromatin accessibility across 36 Level-2 cell classes. Windows overlapping with annotated promoters (TSS ± 2.5 kb) were not excluded, and promoter accessibility was not zeroed.

b, Same as panel **a**, but with promoter accessibility zeroed prior to training (297,936 training windows, 107,014 fine-tuning windows).

c, Same as panel **a**, but with annotated promoter windows (TSS ± 2.5 kb) excluded and promoter accessibility zeroed (299,281 training windows, 106,813 fine-tuning windows). This row corresponds to the primary evolution-naive model referenced throughout the text.

d, Evolution-aware CREsted model trained on 250,902 windows (10,000 most specific peaks per class) drawn from the 354,450 evolutionarily coherent, non-promoter 100-bp windows, followed by fine-tuning on 95,628 windows (3,000 most specific peaks per class), to predict chromatin accessibility across 32 non-promoter cell classes. Promoter accessibility was not zeroed during training, and no TRF replacement was performed during prediction. However, windows exhibiting promoter grammar were effectively excluded if assigned to the promoter cluster as shown in **Fig. 4d**.

e, Same as panel **d**, but with promoter accessibility zeroed prior to training (238,538 training windows, 95,139 fine-tuning windows).

f, Same as panel **e**, but with TRF replacement performed during prediction. This row corresponds to the primary evolution-aware model referenced throughout the text.

g, STEAM-v1 model, trained as in panel **f** but incorporating syntenic orthologs across mammals. TRF replacement was performed during prediction.

bioRxiv preprint doi: https://doi.org/10.64898/2026.04.07.717039; this version posted May 27, 2026. The copyright holder for this preprint (which was not certified by peer review) is the author/funder, who has granted bioRxiv a license to display the preprint in perpetuity. It is made available under a CC-BY 4.0 International license.

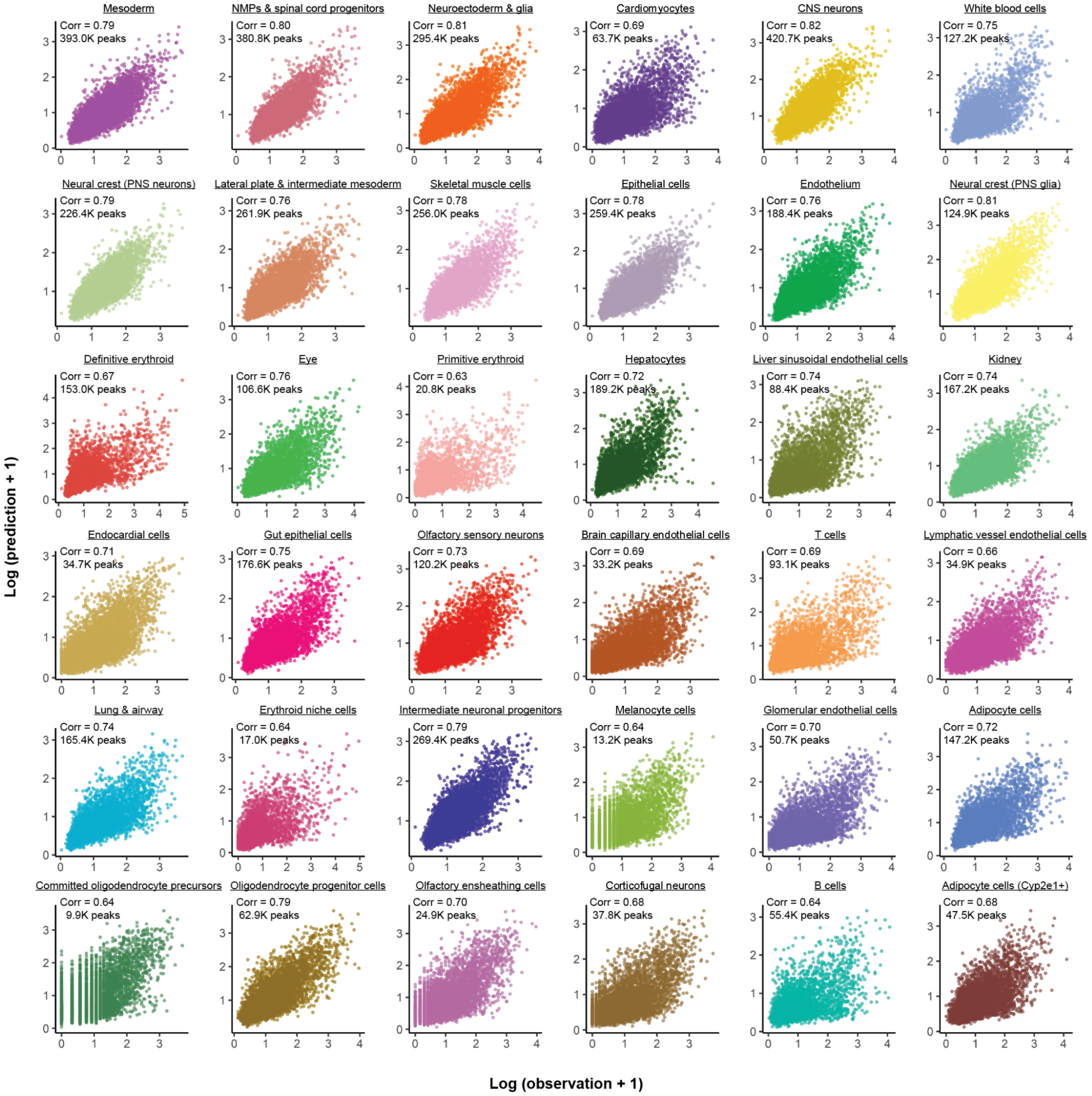

Supplementary Figure 8. Correlation between observed and predicted chromatin accessibility for each cell class. Scatterplots of observed (normalized Tn5 cut-site counts; log-n scaled; *x*-axis) vs. predicted (evolution-naive CREsted model; log-n scaled; *y*-axis) chromatin accessibility across 9,583 held-out peaks held out during fine-tuning, shown separately for each of the 36 Level-2 cell classes. The Pearson's *r* and the total number of peaks called in each cell class (before merging to create a master peak list, but after excluding peaks overlapping the ENCODE blacklist or within 2.5 kb of annotated TSSs) are labeled.

bioRxiv preprint doi: https://doi.org/10.64898/2026.04.07.717039; this version posted May 27, 2026. The copyright holder for this preprint (which was not certified by peer review) is the author/funder, who has granted bioRxiv a license to display the preprint in perpetuity. It is made available under a CC-BY 4.0 International license.

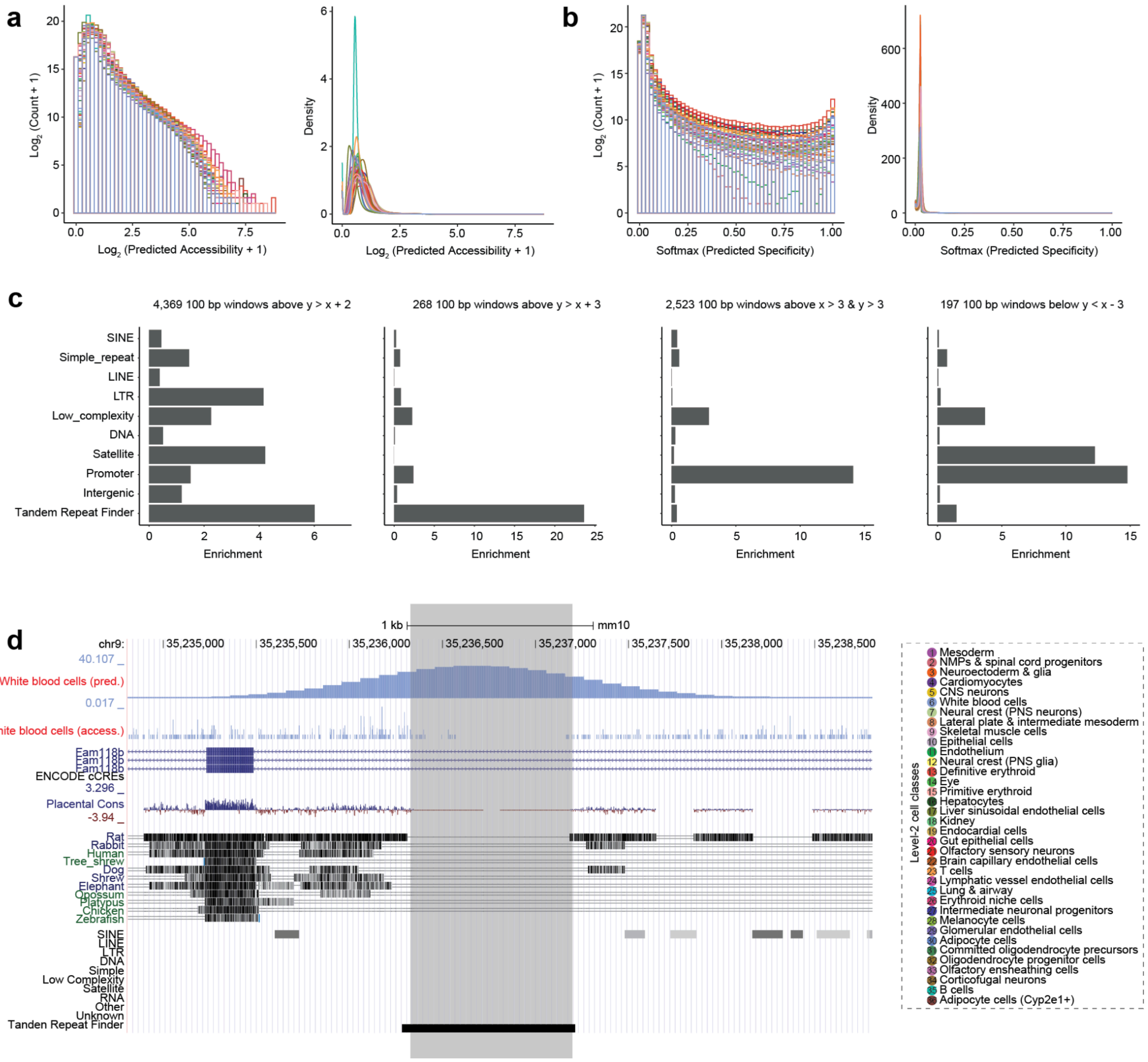

Supplementary Figure 9. Genome-wide, evolution-naive prediction of chromatin accessibility.

a, Histogram (log_2 scaled; left) and density plot (right) of predicted accessibility (log_2 scaled) for 10% (randomly downsampled) of 28M × 100 bp windows spanning the mouse reference genome.

b, Histogram (log_2 scaled; left) and density plot (right) of predicted specificity (log_2 scaled) for 10% (randomly downsampled) of 28M × 100 bp windows spanning the mouse reference genome. Specificity was calculated by applying a softmax function to the predicted accessibility of each window across cell classes.

c, Enrichment of various subsets of evolution-naive predictions spanning mouse chromosome 9 for genome annotations, including (from left to right): moderately or grossly overpredicted ($y > x + 2$), grossly overpredicted ($y > x + 3$), highly accessible and successfully predicted ($x > 3$ and $y > 3$), and grossly underpredicted ($y < x - 3$). Enrichment was quantified as the odds ratio: the fraction of each category overlapping a given annotation, divided by the fraction of chromosome 9 covered by that annotation. For this analysis, promoters were defined as regions within 2.5 kb of annotated TSSs.

d, Genome browser view of observed (normalized Tn5 cut-site counts) and predicted (evolution-naive CREsted model) chromatin accessibility for a representative grossly overpredicted region (highlighted in grey) that overlaps an annotated tandem repeat. Generated using the UCSC Genome Browser (mm10).

bioRxiv preprint doi: https://doi.org/10.64898/2026.04.07.717039; this version posted May 27, 2026. The copyright holder for this preprint (which was not certified by peer review) is the author/funder, who has granted bioRxiv a license to display the preprint in perpetuity. It is made available under a CC-BY 4.0 International license.

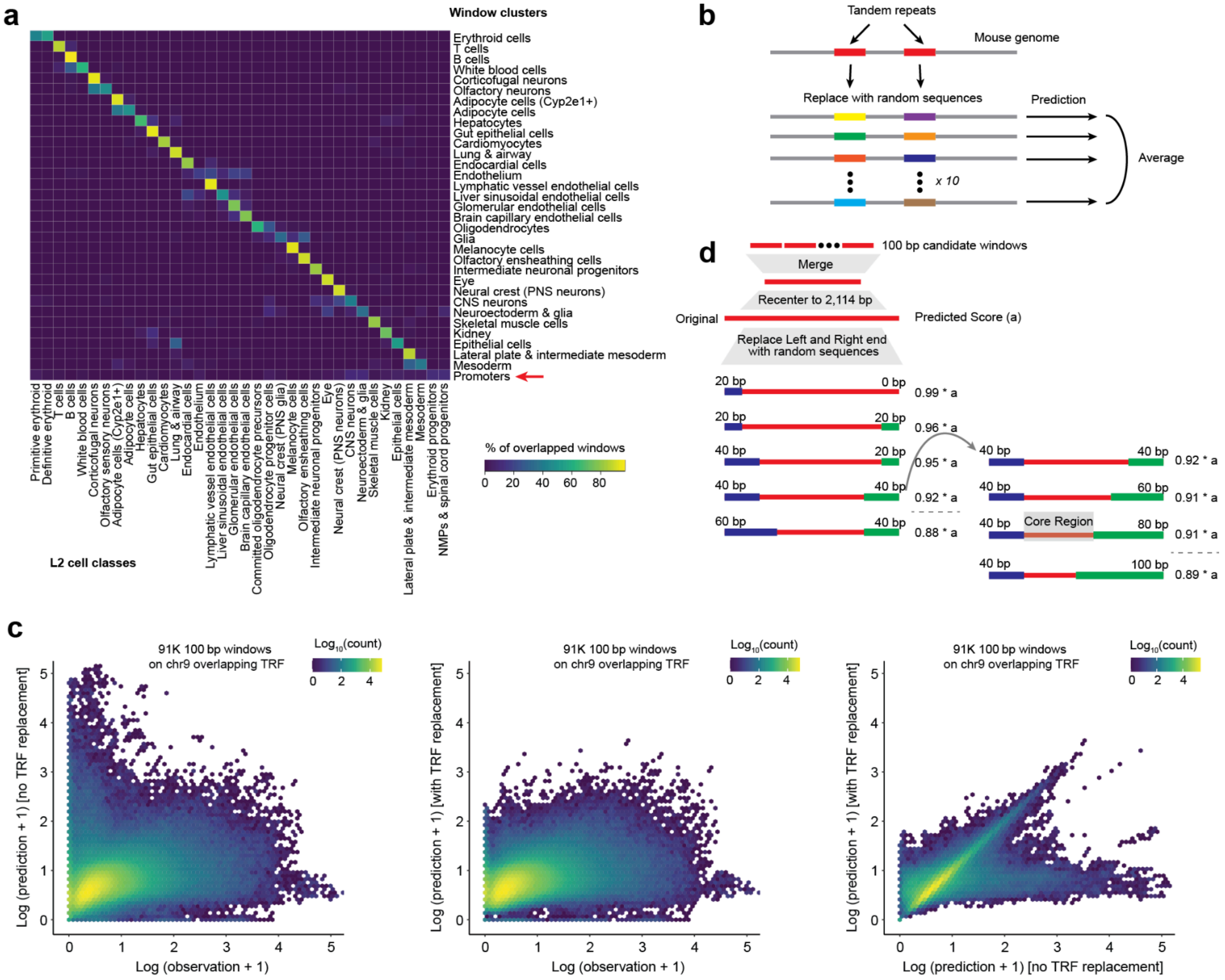

Supplementary Figure 10. Repeat-aware prediction and core-region trimming refine evolution-aware inference.

a, The 547,317 × 100-bp windows that exhibited high evolutionary retention and coherence were subjected to Leiden clustering on their 36-dimensional predicted accessibility vectors, which yielded 33 groups, including 32 cell-class-specific clusters and one large cluster dominated by regions overlapping promoters (row with red arrow). For each of the 33 window clusters (rows), we quantified its intersection with 100-bp windows deriving from each of the 36 Level-2 cell classes (columns). The numbers of overlapping windows were first normalized by the total counts within each column and then converted to percentages for each row. Window clusters and Level-2 cell classes exhibited a largely one-to-one mapping, with a few exceptions as discussed in the text.

b, We performed genome-wide prediction using the evolution-aware model. For this, as with genome-wide prediction using the evolution-naive model, the mouse genome was segmented into sliding 1,000 bp windows, and predictions averaged to 100 bp resolution. However, to mitigate false positive overpredictions induced by tandem repeats, we implemented a heuristic in the mouse reference genome (mm10) was modified by replacing tandem repeats, identified by Tandem Repeats Finder[98] (TRF), with nearly random sequences that matched to local dinucleotide frequency (± 2,500 bp from the center position of each repeat). We repeated this procedure 10 times (*i.e.* genome-wide prediction on 10 modified versions of the mm10 reference genome) and took the mean for each window as its final prediction score.

bioRxiv preprint doi: https://doi.org/10.64898/2026.04.07.717039; this version posted May 27, 2026. The copyright holder for this preprint (which was not certified by peer review) is the author/funder, who has granted bioRxiv a license to display the preprint in perpetuity. It is made available under a CC-BY 4.0 International license.

c, Impact of the tandem repeat overprediction mitigation heuristic. Left: Scatterplots of observed (normalized Tn5 cut-site counts; log-n scaled; *x*-axis) vs. predicted (evolution-aware CREsted model without the tandem repeat mitigation heuristic; log-n scaled; *y*-axis) chromatin accessibility across ~91,000 × 100 bp windows from mouse chr. 9 overlapping TRF-annotated[98] tandem repeats, for all 32 cell classes. Middle: Same as left panel, but with the tandem repeat mitigation heuristic**.** Right: Scatterplots comparing predicted chromatin accessibility of these ~91,000 × 100 bp windows without (*x*-axis) vs. with (*y*-axis) the tandem repeat mitigation heuristic.

d, Schematic of trimming heuristic used to identify "core regions" driving high scores. For each of the 32 cell classes, we selected 100-bp windows with a GPS score > 24.5 (n = ~100,000; ~10 Mb in aggregate) and merged adjacent windows. For each merged region, we extracted a 2,114-bp sequence (the CREsted model input length) centered on the region. We then iteratively replaced increasing amounts of sequence from the left and right ends with random DNA matched to local dinucleotide frequencies (± 2,500 bp from the center), following the pattern [20,0] → [20,20] → [40,20] → [40,40] → …, where each pair indicates the number of base pairs replaced at the left and right ends. At each step, both replaced sequence blocks were regenerated using newly sampled random DNA, and accessibility was re-predicted by the model. When the predicted score first fell below 90% of the original value, trimming from the end whose replacement block had most recently increased was halted, while trimming continued from the opposite end until it also caused the score to drop below the threshold. The core region was defined as the subsequence from immediately before the halt-inducing trim.

bioRxiv preprint doi: https://doi.org/10.64898/2026.04.07.717039; this version posted May 27, 2026. The copyright holder for this preprint (which was not certified by peer review) is the author/funder, who has granted bioRxiv a license to display the preprint in perpetuity. It is made available under a CC-BY 4.0 International license.

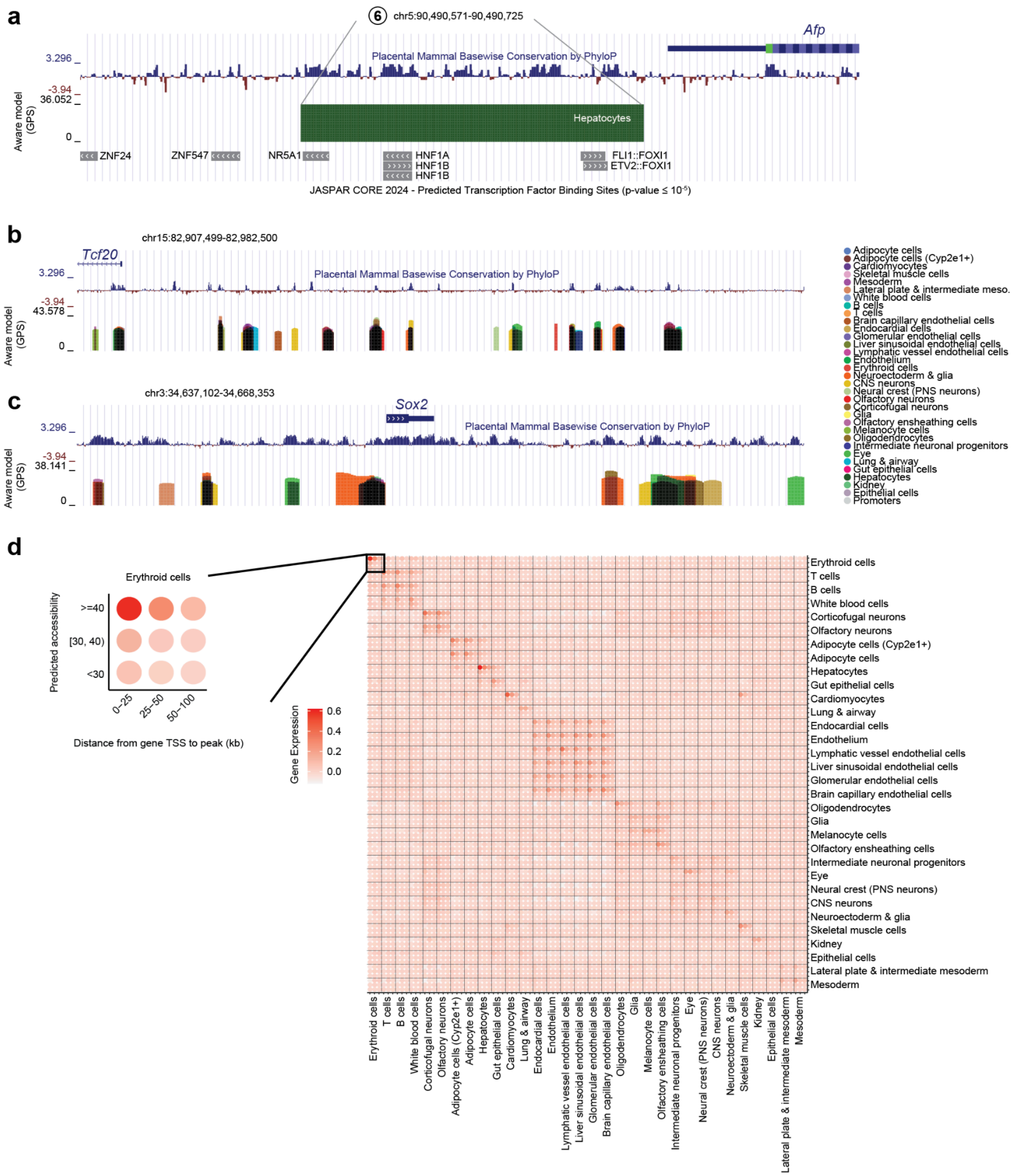

Supplementary Figure 11. Evolution-aware modeling predicts complex regulatory landscapes near developmental transcription factors.

a, Detailed genome browser view of candidate hepatocyte enhancer #6 (chr5:90,490,571-90,490,725), plus 100 bp upstream and downstream. Tracks show phyloP conservation, evolution-aware predicted accessibility (GPS-scaled and trimmed to core region) across 32 cell classes (colors), and JASPAR transcription factor binding motif predictions ($p \leq 1\times10^{-5}$). The element lies immediately upstream of the *Afp* TSS and is selectively predicted in hepatocytes (green), consistent with either an immediately proximal enhancer or cell-type-specific promoter.

bioRxiv preprint doi: https://doi.org/10.64898/2026.04.07.717039; this version posted May 27, 2026. The copyright holder for this preprint (which was not certified by peer review) is the author/funder, who has granted bioRxiv a license to display the preprint in perpetuity. It is made available under a CC-BY 4.0 International license.

b, Genome browser view of the region upstream of *Tcf20* (chr15:82,907,499-82,982,500). Tracks show phyloP conservation and evolution-aware predicted accessibility (GPS-scaled and trimmed to core region) across 32 cell classes (colors). Cell class labels are shown on the right. This region contains predicted enhancers with diverse cell-class-specificities.

c, Same as panel **b** but for the region encompassing *Sox2* (chr3:34,637,102-34,668,353). This region also contains predicted enhancers with diverse cell-class-specificities.

d, For each pairwise cluster comparison, gene-enhancer pairs were binned into a 3 × 3 grid by genomic distance (*x*-axis) evolution-aware predicted accessibility (GPS-scaled), as highlighted to the left (erythroid cells × erythroid cells). Within each bin of the 3 × 3 grid, the mean cell-type-specific expression was computed and background-corrected using 100 permutations with shuffled peak-cell type assignments.

bioRxiv preprint doi: https://doi.org/10.64898/2026.04.07.717039; this version posted May 27, 2026. The copyright holder for this preprint (which was not certified by peer review) is the author/funder, who has granted bioRxiv a license to display the preprint in perpetuity. It is made available under a CC-BY 4.0 International license.

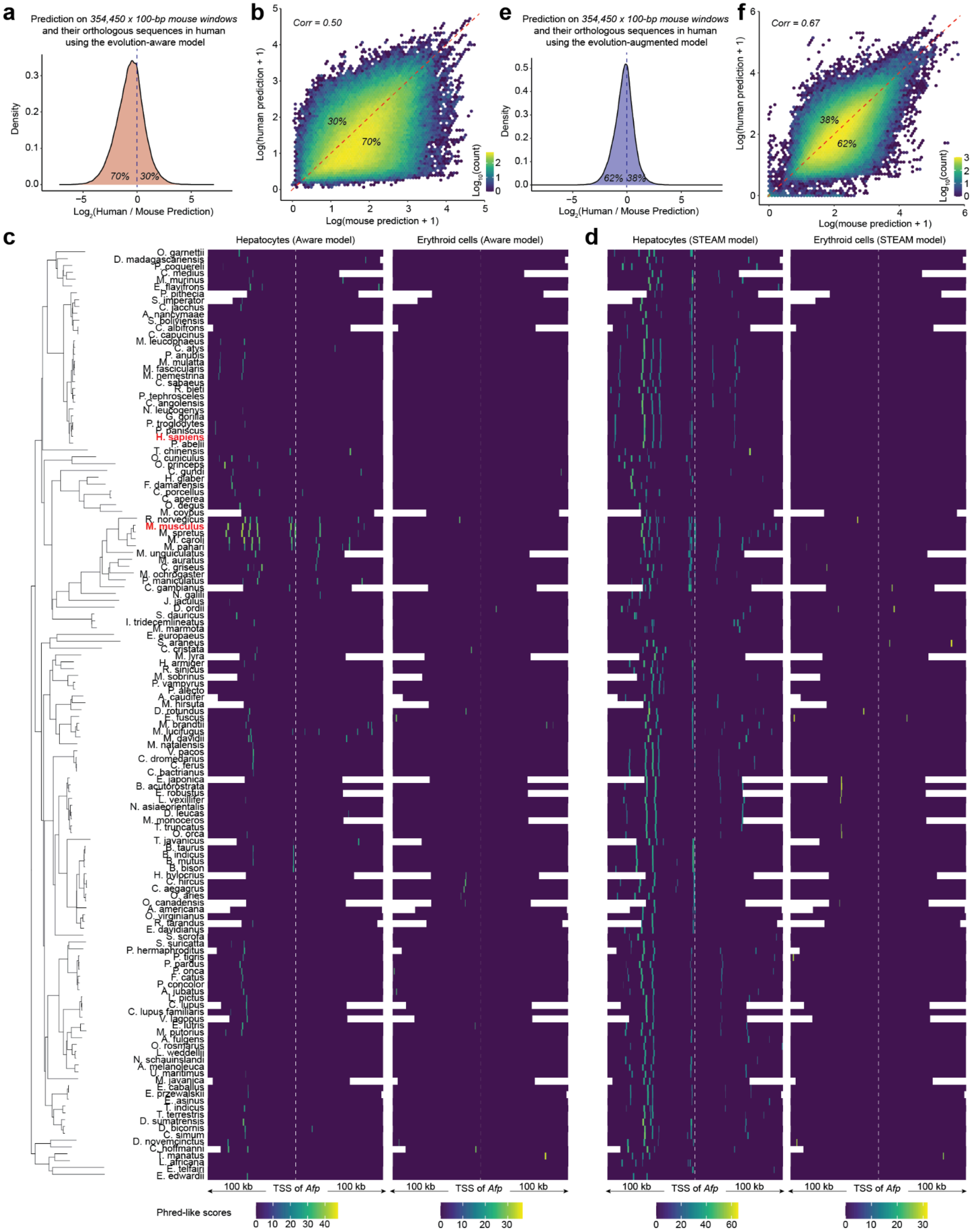

Supplementary Figure 12. Evolution-augmented modeling improves cross-species inference.

a, A set of 354,450 × 100-bp mouse windows were lifted over to the human genome. Both the original windows in the mouse genome and the syntenic regions in the human genome were then extended to 2,114 bp and predicted using the

bioRxiv preprint doi: https://doi.org/10.64898/2026.04.07.717039; this version posted May 27, 2026. The copyright holder for this preprint (which was not certified by peer review) is the author/funder, who has granted bioRxiv a license to display the preprint in perpetuity. It is made available under a CC-BY 4.0 International license.

evolution-naive model. The density of log_2-scaled fold-differences between predicted chromatin accessibility for human vs. mouse across 288,982 syntenic pairs is plotted for all 32 cell classes.

b, Scatterplots show predicted chromatin accessibility in mouse (evolution-aware CREsted model together with tandem repeat mitigation heuristic; log-n scaled; *x*-axis) vs. predicted chromatin accessibility in human (same strategy; *y*-axis) across 288,982 syntenic pairs, for all 32 cell classes, with points colored by log_{10}-scaled density.

c, We examined predicted chromatin accessibility using the evolution-aware model across a 200 kb region centered on the *Afp* TSS (chr5: 90,390,737–90,590,737). The mouse *Afp* TSS was lifted over to other mammalian genomes, and ±100 kb of surrounding sequence was extracted. We retained 136 species with at least 50 kb of contiguous recoverable sequence on each side. Contigs harboring the *Afp* TSS were reoriented to match the top-strand (forward) orientation of the *Afp* transcript in mm10. For each syntenic locus, predictions were performed with tiling, tandem repeat mitigation, and conversion to GPS scores (uniformly calibrated against mouse genome-wide predictions). The left panel shows the phylogenetic tree, restricted to the 136 species included here from the full set of 241 Zoonomia species. The middle and right panels show regions with GPS scores >24.5 from evolution-aware enhancer predictions for hepatocytes (middle) and erythroid cells (right).

d, Similar to panel **c**, but with predictions made using the STEAM-v1 model instead of the evolution-aware model.

e, Similar to panel **a**, but with predictions made using the STEAM-v1 model instead of the evolution-aware model.

f, Similar to panel **b**, but with predictions made using the STEAM-v1 model instead of the evolution-aware model.

bioRxiv preprint doi: https://doi.org/10.64898/2026.04.07.717039; this version posted May 27, 2026. The copyright holder for this preprint (which was not certified by peer review) is the author/funder, who has granted bioRxiv a license to display the preprint in perpetuity. It is made available under a CC-BY 4.0 International license.

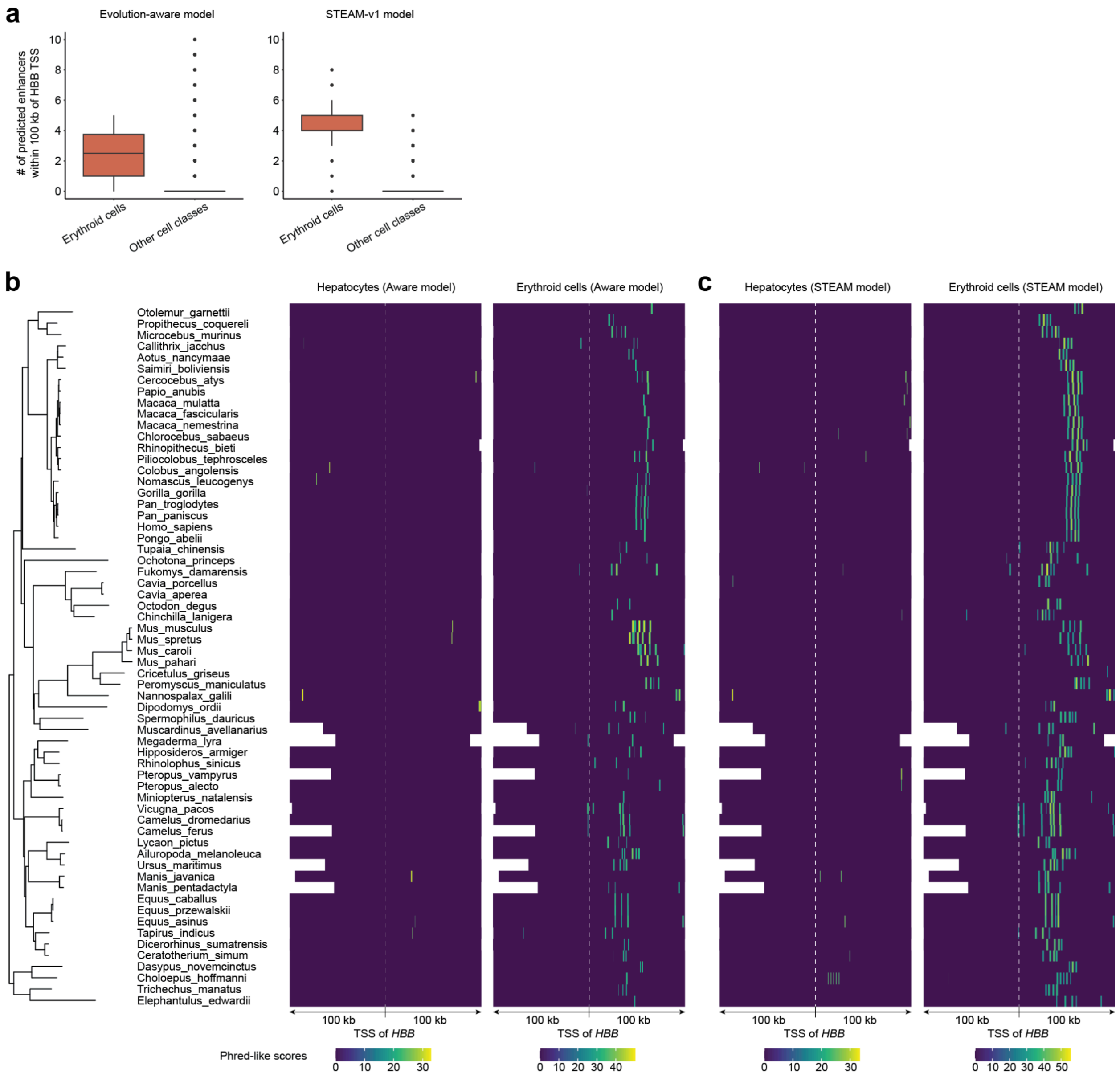

Supplementary Figure 13. Enhancer prediction at the β-globin locus control region in 62 mammalian genomes.

a, The human *HBB* TSS was lifted over to 240 Zoonomia[3] genomes and ±100 kb extracted. 62 species with ≥50 kb of contiguous recoverable sequence on each side were retained. In the human genome, this region includes the classic β-globin locus control region (LCR). The evolution-naive (left) and STEAM-v1 (right) models were applied to each syntenic locus (tiling, tandem repeat mitigation, GPS-based enhancer calling calibrated on mouse genome-wide scale, model-based trimming), and erythroid enhancer counts per species compared against the other 31 cell classes. Boxplot center lines: medians; box limits: 25th–75th percentiles. While the evolution-aware model predicts 2.5 ± 1.4 erythroid enhancers per species (0.6 ± 1.9 for other cell classes), STEAM-v1 predicts 4.2 ± 1.3 (0.2 ± 0.6 for other cell classes).

b, We examined predicted chromatin accessibility throughout this *HBB* TSS centered region. Contigs harboring the *HBB* TSS were reoriented to match the top-strand (forward) orientation of the *HBB* transcript in hg38. The left panel shows the phylogenetic tree, restricted to the 62 species included here from the full set of 241 Zoonomia species. The middle and right panels show regions with GPS scores >24.5 from evolution-aware enhancer predictions for hepatocytes (left) and erythroid (right) cell classes.

c, Similar to panel **b**, but with predictions made using the STEAM-v1 model instead of the evolution-aware model.

bioRxiv preprint doi: https://doi.org/10.64898/2026.04.07.717039; this version posted May 27, 2026. The copyright holder for this preprint (which was not certified by peer review) is the author/funder, who has granted bioRxiv a license to display the preprint in perpetuity. It is made available under a CC-BY 4.0 International license.

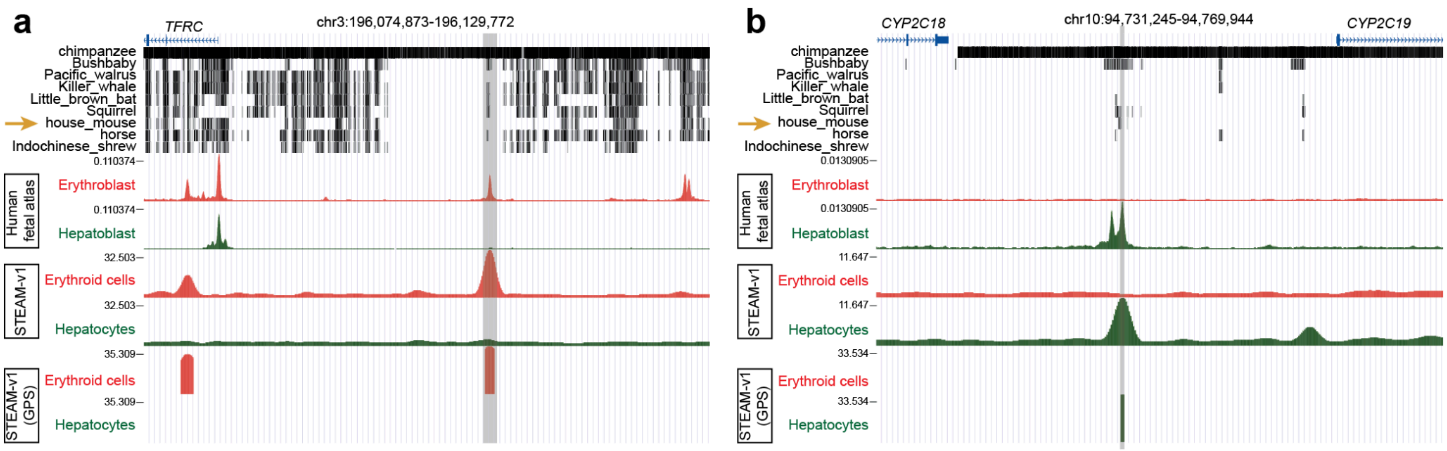

Supplementary Figure 14. Human-specific enhancers at the TFRC and CYP2C19 loci lack syntenic mouse orthologs.

a, Genome browser view of a human erythroid-specific enhancer with no syntenic mouse ortholog, located ~26 kb upstream of the *TFRC* TSS. Tracks show (top to bottom): Zoonomia 241-species alignment[3], observed liver erythroblast and hepatoblast accessibility from the human fetal atlas[53], STEAM-v1 predicted erythroid and hepatocyte accessibility, and GPS-scaled core enhancer predictions. Generated using the UCSC Genome Browser (hg38).

b, Same as panel **a** for a human hepatocyte-specific enhancer with no syntenic mouse ortholog, located ~15 kb upstream of the *CYP2C19* TSS, in the intergenic region between *CYP2C18* and *CYP2C19*.

bioRxiv preprint doi: https://doi.org/10.64898/2026.04.07.717039; this version posted May 27, 2026. The copyright holder for this preprint (which was not certified by peer review) is the author/funder, who has granted bioRxiv a license to display the preprint in perpetuity. It is made available under a CC-BY 4.0 International license.

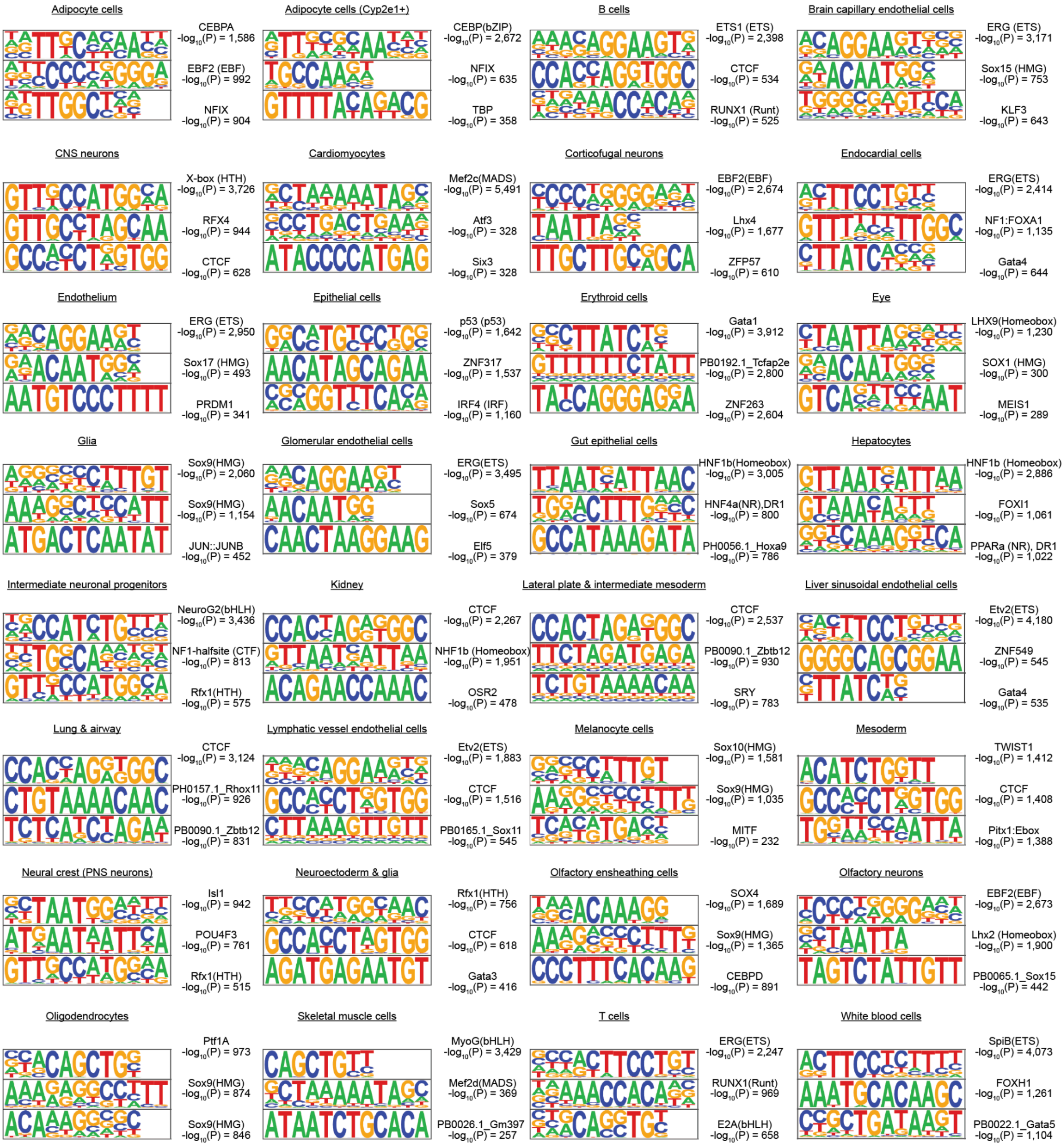

Supplementary Figure 15. Top enriched TF motifs from STEAM-v1 predicted mouse enhancers across 32 cell classes. The top three *de novo* transcription factor binding motifs (ranked by p-value), along with their p-values and best-matched known transcription factor binding motifs, are shown for each cell class. Motifs were identified from the candidate mouse developmental enhancers predicted by the STEAM-v1 model in each cell class using HOMER[100] with the findMotifsGenome.pl function.

bioRxiv preprint doi: https://doi.org/10.64898/2026.04.07.717039; this version posted May 27, 2026. The copyright holder for this preprint (which was not certified by peer review) is the author/funder, who has granted bioRxiv a license to display the preprint in perpetuity. It is made available under a CC-BY 4.0 International license.

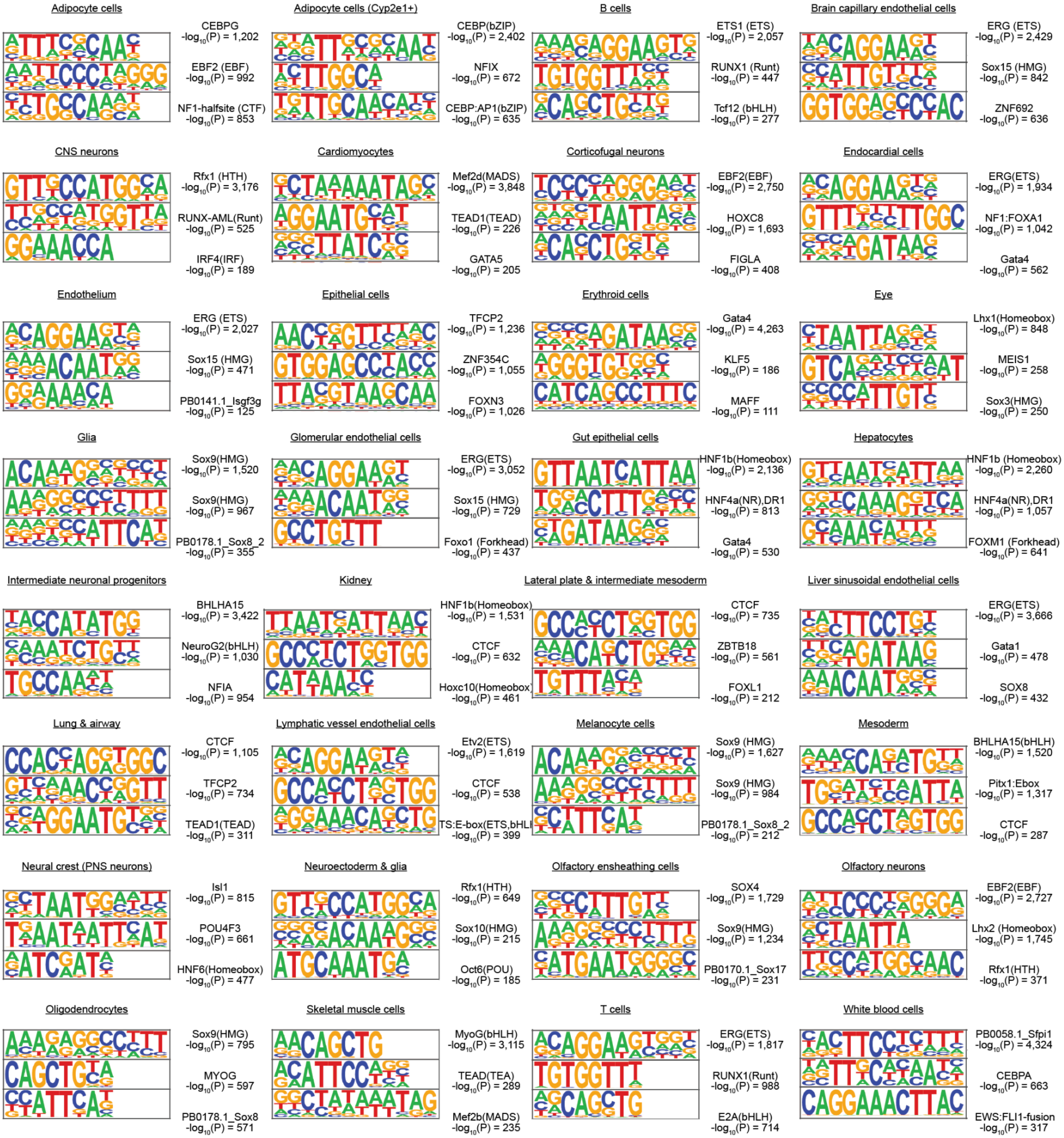

Supplementary Figure 16. Top enriched TF motifs from STEAM-v1 predicted human enhancers across 32 cell classes. The top three *de novo* transcription factor binding motifs (ranked by p-value), along with their p-values and best-matched known transcription factor binding motifs, are shown for each cell class. Motifs were identified from the candidate human developmental enhancers predicted by the STEAM-v1 model in each cell class using HOMER[100] with the findMotifsGenome.pl function.
